# Mirrored STDP Implements Autoencoder Learning in a Network of Spiking Neurons

**DOI:** 10.1371/journal.pcbi.1004566

**Published:** 2015-12-03

**Authors:** Kendra S. Burbank

**Affiliations:** Department of Statistics, University of Chicago, Chicago, Illinois, United States of America; Université Paris Descartes, Centre National de la Recherche Scientifique, FRANCE

## Abstract

The autoencoder algorithm is a simple but powerful unsupervised method for training neural networks. Autoencoder networks can learn sparse distributed codes similar to those seen in cortical sensory areas such as visual area V1, but they can also be stacked to learn increasingly abstract representations. Several computational neuroscience models of sensory areas, including Olshausen & Field’s Sparse Coding algorithm, can be seen as autoencoder variants, and autoencoders have seen extensive use in the machine learning community. Despite their power and versatility, autoencoders have been difficult to implement in a biologically realistic fashion. The challenges include their need to calculate differences between two neuronal activities and their requirement for learning rules which lead to identical changes at feedforward and feedback connections. Here, we study a biologically realistic network of integrate-and-fire neurons with anatomical connectivity and synaptic plasticity that closely matches that observed in cortical sensory areas. Our choice of synaptic plasticity rules is inspired by recent experimental and theoretical results suggesting that learning at feedback connections may have a different form from learning at feedforward connections, and our results depend critically on this novel choice of plasticity rules. Specifically, we propose that plasticity rules at feedforward versus feedback connections are temporally opposed versions of spike-timing dependent plasticity (STDP), leading to a symmetric combined rule we call Mirrored STDP (mSTDP). We show that with mSTDP, our network follows a learning rule that approximately minimizes an autoencoder loss function. When trained with whitened natural image patches, the learned synaptic weights resemble the receptive fields seen in V1. Our results use realistic synaptic plasticity rules to show that the powerful autoencoder learning algorithm could be within the reach of real biological networks.

## Author Summary

In the brain areas responsible for sensory processing, neurons learn over time to respond to specific features in the external world. Here, we propose a new, biologically plausible model for how groups of neurons can learn which specific features to respond to. Our work connects theoretical arguments about the optimal forms of neuronal representations with experimental results showing how synaptic connections change in response to neuronal activity. Specifically, we show that biologically realistic neurons can implement an algorithm known as autoencoder learning, in which the neurons learn to form representations that can be used to reconstruct their inputs. Autoencoder networks can successfully model neuronal responses in early sensory areas, and they are also frequently used in machine learning for training deep neural networks. Despite their power and utility, autoencoder networks have not been previously implemented in a fully biological fashion. To perform the autoencoder algorithm, neurons must modify their incoming, feedforward synaptic connections as well as their outgoing, feedback synaptic connections—and the changes to both must depend on the errors the network makes when it tries to reconstruct its input. Here, we propose a model for activity in the network and show that the commonly used spike-timing-dependent plasticity paradigm will implement the desired changes to feedforward synaptic connection weights. Critically, we use recent experimental evidence to propose that feedback connections learn according to a temporally reversed plasticity rule. We show mathematically that the two rules combined can approximately implement autoencoder learning, and confirm our results using simulated networks of integrate-and-fire neurons. By showing that biological neurons can implement this powerful algorithm, our work opens the door for the modeling of many learning paradigms from both the fields of computational neuroscience and machine learning.

## Introduction

Neurons in the brain’s sensory areas need to form useful internal representations of the external world. Over the course of development, as these neurons create and modify their synaptic connections, they develop receptive fields which allow them to respond to characteristic stimulus features. The preferred features are relatively simple for neurons in primary areas such as primary visual cortex (V1) and primary auditory cortex (A1), but increase in complexity, sparsity, abstractness, and size in higher brain areas. It is an intriguing possibility that the brain uses a similar mechanism to learn receptive fields in higher sensory areas as it does in the primary areas. If so, that mechanism must be flexible enough to work across the different regimes of sparsity, complexity, and abstraction. The mechanism must also be capable of producing representations which are potentially “stackable”, so that the output from one area can be represented in more abstract form in the subsequent area. For instance, if pairwise or higher order correlations in neuronal activity are present in one area, those correlations might be captured to form a more abstract representation in the next area. Finally, the mechanism must be implementable by biological neurons: all computations must be local, and synaptic weight changes should match experimentally observed synaptic plasticity. Here, we introduce a model for learning in a single area which we argue fulfills these requirements: it is biologically plausible while allowing varying levels of sparsity and producing representations that need not be uncorrelated.

Many previous biologically plausible models of receptive field development learn local or “one-hot” representations, in which each stimulus causes approximately *one* neuron (or one small neighborhood of neurons) to respond; models in this class include Kohonen’s Self-Organizing Map [1], LISSOM [2, 3], and Winner-Take-All models [4, 5]. Learning in these models moves the winning neuron’s receptive field closer to the current stimulus using procedures which are simple, synaptically local, and do not require feedback connections. However, local representations have very limited capacity: they can represent O(N) distinct inputs with *N* neurons, thus requiring that the number of neurons is comparable to the number of features to be distinguished. Local models of low-level vision can succeed because natural image patches seem to exist in a space of low dimensionality [6], and spatially localized features can be characterized using only a few parameters (such as orientation, spatial frequency, and phase). However, in higher brain areas with larger and more complex receptive fields, the number of neurons required for a local model to be able to represent all possible stimuli would grow tremendously.

By contrast, distributed models can represent many more potential inputs, from $O(\left(\begin{array}{c} n\\ k \end{array}\right))$ for sparse models with *k* active units up to $O(2^{N})$ for dense models [7–9], and may therefore be better suited for modeling at all levels of the sensory hierarchy. Several biologically plausible models have been proposed for learning distributed representations in the special case where neuronal activity is very sparse and uncorrelated [7, 10–12]; under these conditions, a simple learning rule similar to that seen in the local models can be used. However, a model which does not require neurons to be uncorrelated is desirable because neurons in real cortical networks respond to stimuli in highly correlated ways. This stimulus-dependent correlation should be distinguished from noise correlation, which measures the similarities of *fluctuations* in neuronal responses to identical stimuli. Noise correlation is frequently measured to be small, and so cortical firing is often described as “decorrelated” (e.g. [13]). However, stimulus-dependent correlation is strong; in V1, from 20–50% of neurons have been estimated to respond to each stimulus in their receptive field [14]. Many pairs of neurons have highly correlated responses when measured across multiple stimuli (e.g. [15]). Importantly, in the context of a hierarchy, the correlations remaining in the neurons of one layer can be captured by neurons in subsequent layers.

Perhaps the most well-known model for learning in V1 is Olshausen and Field’s Sparse Coding model [16]. Their algorithm attempts to find receptive fields which simultaneously preserve information while maintaining sparse neuronal activity, but it does not require neuronal activity to be uncorrelated in order to function. However, the algorithm thus far lacks a biological interpretation. A different spike-based matching pursuit model [17] uses different interactions to determine the neuronal activities but the same learning rule, and that learning rule similarly lacks a biological interpretation.

Here, we introduce a novel biological mechanism for a well-known learning algorithm known as the autoencoder. Autoencoders are two-layer neural networks which attempt to learn distributed representations that can be used to accurately reconstruct their inputs. In an autoencoder, external stimuli induce activity in the lower-layer “visible” units. This activity, combined with feedforward connections, then creates a pattern of activity in the upper-layer “hidden” units. Finally, the network uses symmetric or “tied” feedback weights in order to create an attempted reconstruction in the visible layer. The objective of autoencoder learning is to find weights such that the reconstruction closely matches the original stimulus input, thus ensuring that the hidden unit representation is a good one; intuitively, reconstructions can only be accurate when the hidden layer retains sufficient information about the visible layer. An autoencoder can be made to find an *efficient* representation by adding a constraint on the activity or architecture of the hidden layer. This forces the network to find features which are useful for describing the particular types of stimuli seen during training. The constraint can take the form of a regularization term added to the loss function. Alternatively, it can be a hard limit, such as a limit on the number of hidden units, a requirement that hidden units be binary, or a requirement that hidden unit activity be sparse. Typically, autoencoders are trained using stochastic gradient descent on the squared reconstruction error (or on the reconstruction error plus regularizer term); for each stimulus presentation, synaptic weights are changed in the direction that would most decrease this loss function. In this work, networks are trained instead using the “autoencoder rule”, also known as Oja’s subspace rule [18], which is an approximation to the full gradient descent expression. If the vector of input values is given by $\vec{x}$, hidden unit activities are given by $\vec{y}$, and the attempted reconstruction is the vector $\hat{\vec{x}}$, then the autoencoder rule states that for learning rate *η*, the change in synaptic weights *w*_*ij*_ between visible unit *i* and hidden unit *j* is given by

$$\Delta w_{ij}=\eta \left(x_{i}-{\hat{x}}_{i}\right)y_{j}\;\text{(Autoencoder}\;\text{learning}\;\text{rule)}$$ (1)

Autoencoders can be used to accurately model responses in early sensory areas; indeed, Olhausen & Field’s Sparse Coding network is an autoencoder with lateral interactions between the hidden units used to impose a sparsity constraint. But the autoencoder is a very general algorithm. With different neuronal activation functions and lateral interactions, autoencoders can also find the subspace spanned by Principal Component Analysis (PCA) eigenvectors [9, 18] or perform an online implementation of K-means clustering [19]. (See [9] for an extensive review of autoencoders and their relationship to other learning algorithms.) These cases show that autoencoders can span the range between learning dense distributed models, as in PCA [20], and local models, as in K-means. Sparse Coding, where several hidden units respond to each stimulus, falls in between these two extremes. Autoencoders have been used extensively in the machine learning community, where they have been stacked to form multi-layer representations of increasing abstraction [21–23] or used to pre-train deep neural networks that perform classification tasks [24].

There are two main difficulties regarding a biologically plausible implementation of the autoencoder. The first challenge arises from the fact that learning must depend on the *difference* of two neuronal activities: the original visible unit activity $\vec{x}$ and the reconstructed activity $\hat{\vec{x}}$ (see Eq (1)). The second difficulty comes from the required symmetry of learning tied weights, where feedforward weights are equal to feedback weights. Preserving this symmetry over the course of learning dictates that any change to the feedforward synaptic strength between two neurons must be accompanied by an identical change to the feedback strength. If a feedforward synapse is weakened, the feedback synapse must also be weakened, and vice versa. In real neurons, feedforward and feedback synapses are physically distinct entities, and a biologically realistic model must account for how the two can experience identical (or very similar) plasticity.

Previous implementations have addressed these two challenges by positing that hidden layer neurons are inhibitory and create negative reconstructions, so that the final activity in the visible layer is $\vec{\epsilon }=\vec{x}-\hat{\vec{x}}$, and stipulating that learning then proceeds according to symmetric Hebbian rules Δ*w*_*ij*_ = *ϵ*_*i*_ *y*_*j*_ [25, 26]. However, these implementations are biologically unrealistic in three important ways. First, they require visible unit activity levels $\vec{\epsilon }$ to become *negative* at times in order to create synaptic depression. Second, the inhibitory nature of the feedback connections is unrealistic, since it is known that most feedback connections between cortical areas arise from excitatory neurons, and most feature-selective neurons are excitatory [27]. Third, the learning rules themselves are unrealistic; experiments have shown that in real neurons, unlike those modeled in inhibitory feedback networks, synaptic plasticity is neither purely Hebbian nor symmetric. Instead, the sign of synaptic plasticity often depends on the relative timing of activity in pre- and post-synaptic neurons [28, 29], in a process known as spike-timing dependent plasticity (STDP) [29–32].

Here, we instead propose a spiking neural network in which feedback creates a weak, *positive* reconstruction. Unlike a previous proposal with a similar architecture [33], our model uses a biologically realistic synaptic plasticity rule to implement learning. The required negative sign in the learning rule arises naturally from an additive version of STDP, while our proposed differences in the plasticity rules at feedforward versus feedback synapses [34] lead to effective symmetry in learning. We show analytically that the learning in our network approximates the autoencoder learning rule.

To examine the behavior of our model in the sparse regime, we use a very simple, biologically plausible method for inducing individual hidden neurons to have high lifetime sparsity. Our method uses local homeostatic mechanisms within each neuron to drive the network to find sparse solutions, and is designed to mimic a biological process known as “synaptic scaling” [35–37], in which neurons regulate their activity levels by modifying their susceptibility to synaptic inputs. The resulting sparsity is important because it is well known that algorithms which yield sparse representations of natural stimuli can learn synaptic weights which closely resemble the receptive field structures of simple cells in primary sensory cortices (reviewed in [38]). The specific choice of algorithm seems to matter less than its basic ability to create a sparse representation [39]; while Sparse Coding was an early and famous example [16], various well-known sparse algorithms give qualitatively and even quantitatively similar results on visual, auditory, and somatosensory stimuli. These include independent component analysis [40], sparse autoencoders [41], restricted Boltzmann machines [41], and K-means clustering [42] (all are reviewed in [43] and [39]).

We use simulated networks of integrate-and-fire neurons in two experiments to show that our network is capable of minimizing reconstruction error in these example datasets. For the first experiment, in which we train the network using a dataset containing handwritten digits, we use model neurons that approximate the idealized units in a neural network by having synaptic weights that can become positive or negative and an additive form of synaptic scaling that resembles a neural network bias term. For the second experiment, we use a dataset containing whitened natural image patches and we verify that the learned receptive fields resemble those measured in primary visual cortex. Here, we more closely model biological excitatory neurons by restricting synaptic weights to be positive and by using a multiplicative form of synaptic scaling. In both experiments, dynamic parameters such as membrane time constants and synaptic transmission delays are set to biologically realistic values.

## Results

### The autoencoder rule

We begin by defining the general autoencoder problem in a two-layer neural network. Each neuron in the first, visible layer is connected reciprocally to each neuron in the second, hidden layer, and there are no lateral connections. During each training trial, the network is presented with stimulus $\vec{x}$ in the visible layer. The network then computes a representation $\vec{y}$ in the hidden layer, using feedforward weights **W** (with the *j*th column vector denoted by ${\vec{w}}_{j}$) and other parameters *θ* according to the potentially non-linear function

$$y_{j}=f\left(\vec{x};{\vec{w}}_{j},\theta \right).$$ (2)

The network then computes an attempted reconstruction $\hat{\vec{x}}$ in the visible layer using symmetrical or “tied” feedback weights $W^{⊺}$ and an activation function *g*, so that

$$\hat{x_{i}}=g\left(\sum_{j}y_{j}w_{ij}\right).$$ (3)

A squared reconstruction error is defined as

$$E=\|\vec{x}-\hat{\vec{x}}{\|}^{2}=\sum_{i}{\left(x_{i}-{\hat{x}}_{i}\right)}^{2}.$$ (4)

How should this network modify its weights so as to minimize this error, using stochastic gradient descent? The derivative of E with respect to the weights *w*_*ij*_ between a visible neuron *i* and a hidden neuron *j* is

$$\frac{\partial E}{\partial w_{ij}}=2\sum_{i^{\prime }}\left(x_{i^{\prime }}-{\hat{x}}_{i^{\prime }}\right)\left(\frac{\partial x_{i^{\prime }}}{\partial w_{ij}}-\frac{\partial {\hat{x}}_{i^{\prime }}}{\partial w_{ij}}\right).$$ (5)

The initial visible activities *x*_*i*_ don’t depend on the weights, so $\frac{\partial x_{i^{\prime }}}{\partial w_{ij}}=0$ for all *i*′. Similarly, the hidden unit activity *y*_*j*_ is independent of the weights to other hidden units, so $\frac{\partial y_{j^{\prime }}}{\partial w_{ij}}=0$ for *j* ≠ *j*′. If we define

$$g_{i}^{\prime }\equiv \frac{dg}{dz}{|}_{z=\sum_{j}y_{j}w_{ij}}$$ (6)

then

$$\frac{\partial {\hat{x}}_{i^{\prime }}}{\partial w_{ij}}=g_{i^{\prime }}^{\prime }\sum_{j^{\prime }}y_{j^{\prime }}\frac{\partial w_{i^{\prime }j^{\prime }}}{\partial w_{ij}}+g_{i^{\prime }}^{\prime }\sum_{j^{\prime }}\frac{\partial y_{j^{\prime }}}{\partial w_{ij}}w_{i^{\prime }j^{\prime }}=g_{i^{\prime }}^{\prime }y_{j}\delta_{i,i^{\prime }}+g_{i^{\prime }}^{\prime }\frac{\partial y_{j}}{\partial w_{ij}}w_{i^{\prime }j}.$$ (7)

Therefore,

$$-\frac{1}{2}\frac{\partial E}{\partial w_{ij}}=\left(x_{i}-{\hat{x}}_{i}\right)g_{i}^{\prime }y_{j}+\sum_{i^{\prime }}\left(x_{i^{\prime }}-{\hat{x}}_{i^{\prime }}\right)g_{i^{\prime }}^{\prime }\frac{\partial y_{j}}{\partial w_{ij}}w_{i^{\prime }j}.$$ (8)

There are two terms above because of the tied weights: changing *w*_*ij*_ modifies both feedforward and feedback connections, and these changes have two independent effects on the reconstruction error. The first term is simpler, and reflects the contribution from the changed feedback connections. Importantly, it depends only on the activities of the connected neurons *i* and *j*. We therefore say that it is a “local” computation, and one that might plausibly be computed by biological neurons.

By contrast, the second term, which reflects the contribution from the changed feedforward connections, is non-local. It depends on the activities of every visible neuron; this information would not be available to a biological synapse. Previous authors have noted that the second term is often small [44], so that an *approximate* gradient descent using only the first term works nearly as well as the full equation [44, 45]. For linear reconstructions, where *g*′ is a constant, this becomes the autoencoder learning rule (Eq 1). This is the rule that we will implement biologically.

We note that the designation of “local” or “non-local” depends upon the activity in the network. We could have written ${\hat{x}}_{i}$ in Eq (1) as *g*(∑_*j*′_ *y*_*j*′_ *w*_*ij*′_), and the learning rule would have appeared non-local due to its dependence on the *y*_*j*′_ terms. Indeed, it is this exact non-locality that has caused previous authors to argue that autoencoder learning is not biologically plausible (e.g. [12]). Here, instead, information about all hidden-unit activities is incorporated into the reconstruction activations ${\hat{x}}_{i}$ of the visible units themselves. Any synaptic plasticity rule which incorporates ${\hat{x}}_{i}$ will allow synaptic changes to depend on the activity of all the hidden units and the initial activity of all the visible units—even though the learning rule is purely local.

### Spiking network for autoencoder learning

**Fig 1:**
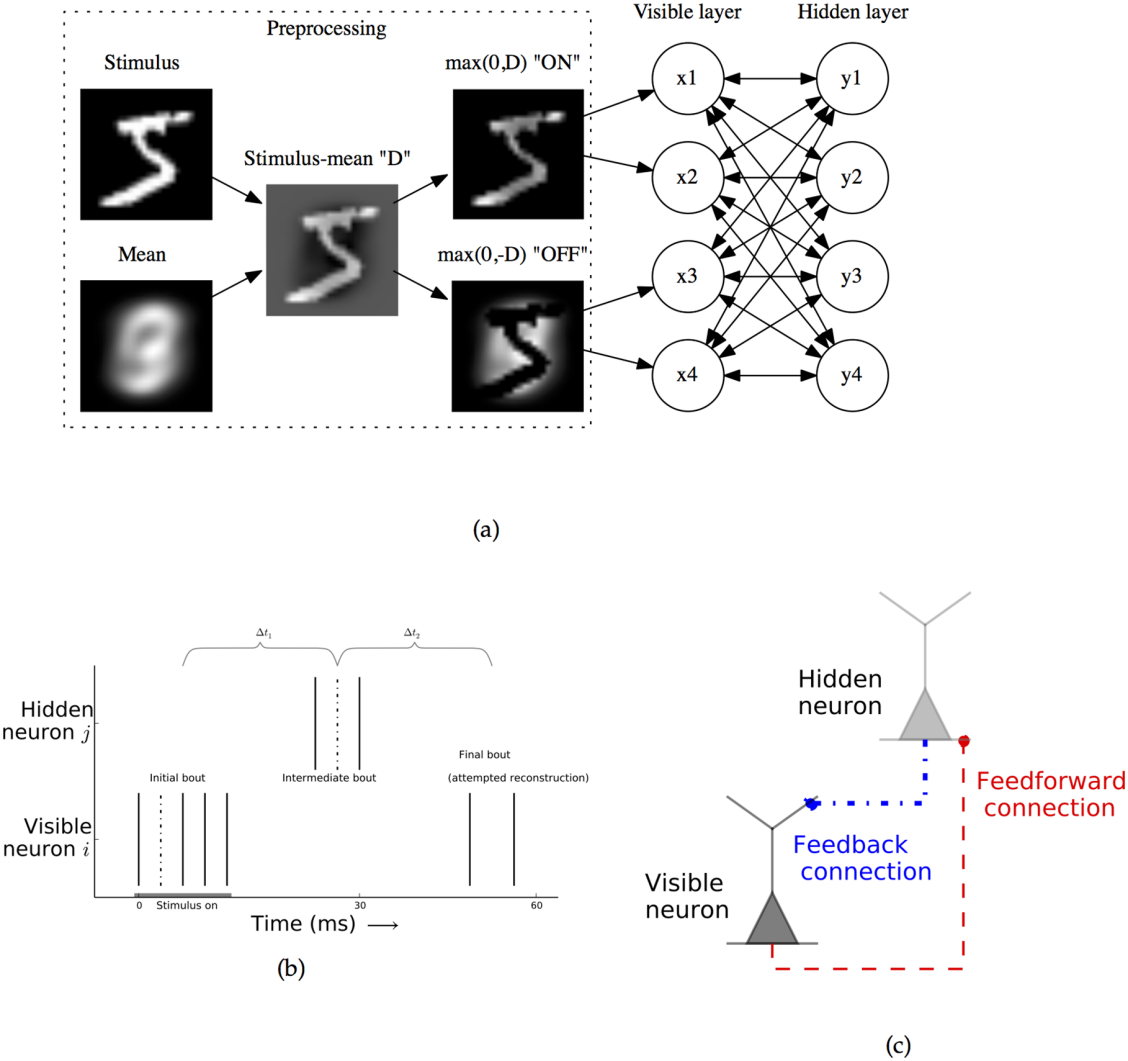
Architecture of the model network and stimulus preprocessing. Architecture of the model network and network activity. **a:** Architecture of the model network and stimulus preprocessing. The final preprocessing step of separating the stimulus into two non-negative “ON” and “OFF” populations allows the visible layer activities to remain positive. **b:** Example activity of two neurons in the spiking network. In response to external stimulus onset (gray bar), the visible neuron *i* fires several spikes in the “initial bout” of activity. After a delay, feedforward excitation causes the hidden neuron *j* to fires spikes in the “intermediate bout”. After another delay, feedback causes the visible neuron to spike in the “final bout”, the network’s attempted reconstruction. The average time between spikes in the initial and intermediate bouts and intermediate and final bouts are given by Δ*t*_1_ and Δ*t*_2_, respectively. Every pair of visible and hidden spikes contributes to plasticity, dependent on their relative times. Learning from two example dotted spikes is described in Fig 2. **c:** Biological feedforward and feedback connections are physically distinct. For the feedforward connection, the visible neuron is pre-synaptic, the hidden neuron is post-synaptic, and the synapse lies close to the hidden neuron’s cell body. For the feedback connection, the hidden neuron is pre-synaptic, the visible neuron post-synaptic, and the synapse is far out on the visible neuron’s dendritic tree.

To implement the autoencoder learning rule with biologically realistic neurons, we propose a two-layer network of spiking neurons with *N*_vis_ neurons in the visible layer and *N*_hid_ neurons in the hidden layer (Fig 1a). Every visible neuron is connected reciprocally with every hidden one, and there are no lateral connections within a layer. The matrix of feedforward connections is denoted **W** and the feedback connection matrix is **Q**; following sections will show how the weights become symmetric.

Inputs to the network are pixel values of preprocessed training images, and they stimulate only the visible neurons (Fig 1a). Plasticity in the system has two components: inter-layer synaptic weights evolve according to the mirrored STDP (mSTDP) rules, and hidden neurons homeostatically adjust synaptic scaling to maintain target average activity levels (both are described below). In our simulations, we use the leaky-integrate-and-fire (LIF) model for the neurons. However, our main results only depend on an approximately linear relationship between input strength and neuronal firing rate, so other neuron models could work as well. The details of the model implementation and all parameters used in the simulations are summarized in S1–S7 Tables.

Our input preprocessing begins with a mean-subtraction step. This leaves pixel values ${\vec{\nu }}_{ext}$ that can be either positive or negative, which allows for a parsimonious representation of input pixels that are above or below their average values. However, biological neurons cannot have negative firing rates. We accommodate this by using an “ON-OFF cell” strategy, which uses twice as many visible neurons as pixels in the stimulus image. The inputs for the first half of the visible neurons are ${\vec{\nu }}_{\text{ON}}=\max\left(0,{\vec{\nu }}_{ext}\right)$, while the inputs to the second half of visible neurons are ${\vec{\nu }}_{\text{OFF}}=\max\left(0,-{\vec{\nu }}_{ext}\right)$. This strategy closely resembles that used by subcortical cells in the mammalian visual system [46, 47], and it allows both the positive and negative areas of the mean-subtracted natural image patches to be represented with positive neuronal activities of similar magnitude.

The network is trained through the sequential presentations of input stimuli. We choose parameters such that activity in the spiking network occurs in three rough bouts. Fig 1b shows activity for one visible and one hidden unit during a presentation. For each stimulus, visible neurons receive a brief pulse of excitatory synaptic input proportional to stimulus strength. This input causes the neurons to generate a series of spikes; the spike counts during this period are represented by the vector $\vec{x}\in Z_{\geq 0}^{N_{\text{vis}}}$. Feedforward synaptic excitation causes some of the hidden units to spike; their spike counts are given by $\vec{y}\in Z_{\geq 0}^{N_{\text{hid}}}$. These hidden-unit spikes occur at a delay with respect to the initial visible unit activity because of a short synaptic transmission delay and because excitation from many spikes is required before the neurons reach threshold. Finally, after a further delay, visible units may spike again due to feedback excitation. The total number of visible spikes occurring due to feedback is $\hat{x}\in Z_{\geq 0}^{N_{\text{vis}}}$. In Fig 1b *x*_*i*_ = 5, *y*_*j*_ = 3, and ${\hat{x}}_{i}=2$; here, the three bouts are temporally separate, but in simulations there can be some overlap.

To prevent reverberating activity from growing exponentially during the course of a trial, we can consider parameters that lead to *weak feedback*, such that the number of spikes in the attempted reconstructions $\hat{x}$ is several times smaller than that in the the initial activities $\vec{x}$ [34, 48]. We denote the constant scaling factor *α* < 1, and say that the network makes a successful reconstruction when $\hat{\vec{x}}\approx \alpha \vec{x}$.

Tied weights along with weak feedback will be maintained when learning rules enforce the relationship $Q=\alpha W^{⊺}$. Because feedforward and feedback synapses occur at physically distinct locations (Fig 1c), we will show separate, biologically plausible plasticity rules for both feedforward and feedback connections and describe how they can maintain this symmetrical relationship.

We define a scaled spiking reconstruction error $L_{\text{spike}}=\left|\right|\vec{x}-\frac{1}{\alpha }\hat{x}{\left|\right|}^{2}$. To determine what synaptic weight changes will decrease this error, we first need to specify how the spike counts depend on the weights. If the neurons in the network behave like standard leaky integrate-and-fire neurons (and time periods are short compared to the membrane time constant), their spike counts will be well approximated by rectified linear functions, so that $\vec{y}\approx \max\left(0,W\vec{x}\right)$ and $\hat{\vec{x}}\approx \max\left(0,\alpha W^{⊺}\vec{y}\right)$. In this case, the first term of the gradient descent expression for *w*_*ij*_ becomes $\left({\hat{x}}_{i}>0\right)\times (x_{i}-\frac{1}{\alpha }{\hat{x}}_{i})y_{j}$. In the common cases where *x*_*i*_ and ${\hat{x}}_{i}$ are both zero or are both nonzero, this gives the approximate gradient descent rule which the network should follow:

$$\Delta w_{ij}=\left(x_{i}-\frac{1}{\alpha }{\hat{x}}_{i}\right)y_{j}\;\text{(Scaled}\;\text{autoencoder}\;\text{rule)}$$ (9)

Our goal will be to show that biologically realistic synaptic plasticity rules used by the neurons in our network can implement this scaled autoencoder rule both for feedforward and feedback connections.

#### Mirrored STDP rule leads to learning of symmetric connections

**Fig 2:**
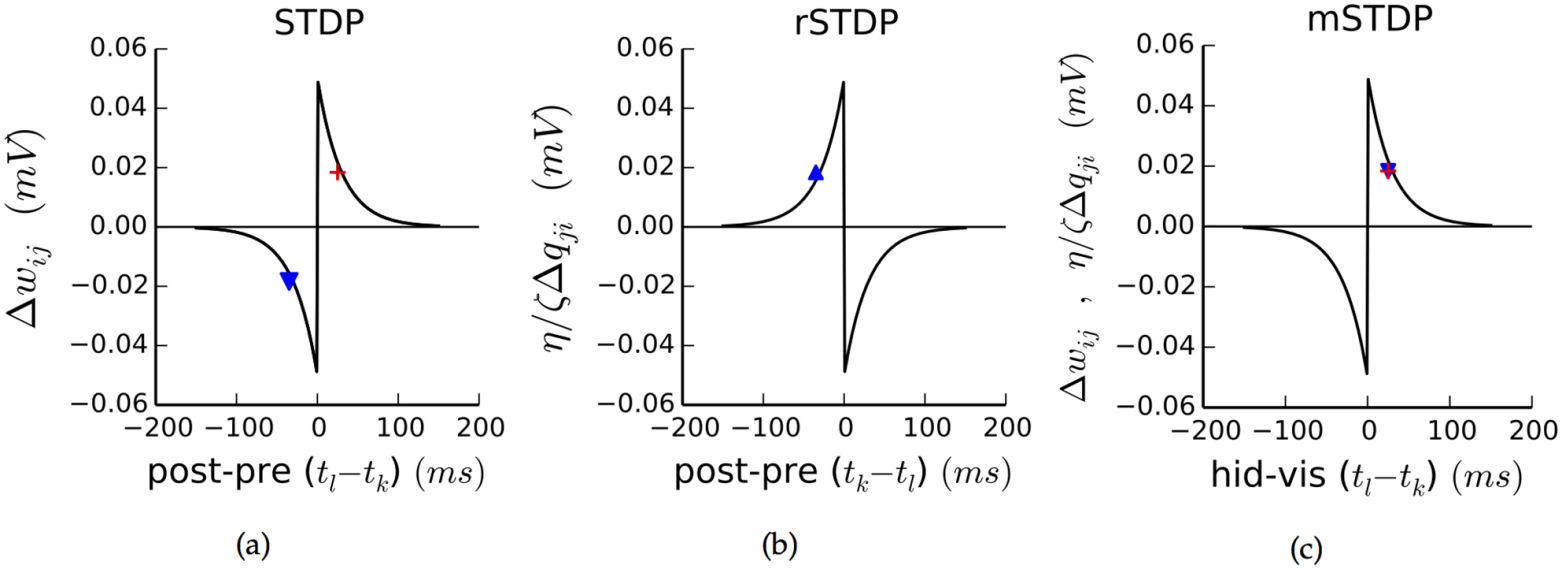
Plasticity rules. Each plot shows plasticity from spikes *k* and *l* from a visible and hidden neuron, respectively, which occur at times *t*_*k*_ and *t*_*l*_. Red cross and blue triangle show learning from the two example dashed spikes in Fig 1b for feedforward and feedback connections, respectively. Note different *x*-axes on each plot. **a:** Standard STDP rule. Used for feedforward connections in the model, for which spike *l* is post-synaptic. *x*-axis shows time difference between post- and pre-synaptic spikes. The example spikes would strengthen the feedforward connection (red cross) but weaken the feedback connection, if feedback followed this rule (blue triangle). **b:** aSTDP rule, in which the time dependence is reversed. Used for feedback connections, for which spike *k* is post-synaptic. Learning rate is scaled by a constant *ζ*/*η* relative to STDP. **c:** Combined mSTDP rule. *x*-axis shows time difference between hidden and visible spikes, leading to identical profiles for STDP and aSTDP. Feedforward and feedback learning is symmetric (red cross and blue triangle).

In our model, feedforward weights between any two neurons *i* and *j* are learned according to the commonly used additive STDP paradigm (Fig 2a), which specifies weight changes due to each pair of spikes in the two neurons. STDP captures the fact that in many biological synapses, the direction of plasticity depends on the relative timing of pre- vs post-synaptic activity [29]. The identities of the pre- and post-synaptic neurons depend on the connection direction: for a connection running from neuron *i* to neuron *j*, neuron *i* is the pre-synaptic one and *j* is post-synaptic. Under STDP, if the pre-synaptic neuron spikes first and is closely followed by a postsynaptic spike, the connection is strengthened. Conversely, if the post-synaptic neuron spikes first, the connection is weakened. The magnitude of the depression or potentiation decreases exponentially with the absolute value of the timing difference. When multiple spikes are fired, the weight change is the sum of the individual change calculated from all possible spike pairs. If $S_{pre}$ and $S_{post}$ are the sets of spikes of the pre- and post-synaptic neurons, respectively, and *t*_*k*_ is the time of the spike *k*, the STDP learning rule is given by:

$$\Delta w_{ij}=\eta \sum_{k\in S_{pre}}\sum_{l\in S_{post}}\left\{\begin{array}{cc} +e^{-|t_{l}-t_{k}|/\tau_{+}} & \text{if}\;t_{l}>t_{k}\\ -e^{-|t_{l}-t_{k}|/\tau_{-}} & \text{if}\;t_{l}\leq t_{k} \end{array}\right.\;\text{(STDP,}\;\text{used}\;\text{for}\;\text{feedforward}\;\text{connections)}\;$$ (10)

Here, *η* is the learning rate and *τ*_+_ and *τ*_−_ are timescales for synaptic potentiation and depression, respectively; for biological synapses, these are typically on the order of tens of milliseconds.

Motivated by two experimental results, we use a slightly different plasticity rule for our feedback connections. First, feedback connections in cortex tend to be at synapses far out on the dendrites of the post-synaptic neuron, unlike feedforward connections which usually arrive close to the cell body (Fig 1c) [27, 49]. Second, in several cortical systems, plasticity at distal synapses has been observed to have a reversed temporal dependence as compared with traditional STDP [50–52]. We have previously postulated [34] that feedback synapses themselves experience anti-Hebbian STDP [53–55], or “aSTDP”, which is temporally reversed compared with standard STDP. With aSTDP, pre-synaptic activity occurring before post-synaptic activity leads to depression, and vice versa. The aSTDP rule is given by (Fig 2b):

$$\Delta q_{ji}=\zeta \sum_{k\in S_{pre}}\sum_{l\in S_{post}}\left\{\begin{array}{cc} +e^{-|t_{l}-t_{k}|/\tau_{+}} & \text{if}\;t_{l}\leq t_{k}\\ -e^{-|t_{l}-t_{k}|/\tau_{-}} & \text{if}\;t_{l}>t_{k} \end{array}\right.\;\text{(aSTDP,}\;\text{used}\;\text{for}\;\text{feedback}\;\text{connections)}$$ (11)

This differs from Eq (10) only in the directions of the greater than/less than signs and in the use of ζ as a learning rate, potentially different from that for STDP.

Critically, we note that for feedforward connections, visible units are pre-synaptic—but the reverse is true for feedback connections (Fig 1c). We can use this fact to update Eq (10) by replacing $S_{pre}$ with the set of visible neuron spikes $S_{i}$, and similarly update Eq (11) by replacing $S_{pre}$ with the set of *hidden* neuron spikes $S_{j}$. After making analogous replacements for $S_{post}$, we see that, up to a constant, the equations have become identical. We call this combined learning rule mSTDP, for mirrored STDP (Fig 2c):

$$\Delta w_{ij}=\frac{\eta }{\zeta }\Delta q_{ji}=\eta \sum_{k\in S_{i}}\sum_{l\in S_{j}}\left\{\begin{array}{cc} +e^{-|t_{l}-t_{k}|/\tau_{+}} & \text{if}\;t_{l}>t_{k}\\ -e^{-|t_{l}-t_{k}|/\tau_{-}} & \text{if}\;t_{l}\leq t_{k} \end{array}\right.\;\text{(mSTDP,}\;\text{for}\;\text{feedforward}\;\text{and}\;\text{feedback}\;\text{connections)}$$ (12)

Thus, under mSTDP the plasticity due to any pair of visible and hidden spikes will be the same for the feedforward connections as for the feedback connections, up to a scaling factor. We note that if we had instead used simple STDP for both feedforward and feedback connections, the plasticity for any pair of spikes would have had the opposite sign for the the two directions—exactly the opposite of the symmetry needed for autoencoder learning (Fig 2a, blue triangle and red cross.)

If the weights are initially symmetric up to a scaling factor, such that $Q=\frac{\eta }{\zeta }W^{⊺}$, mSTDP will maintain that symmetry. Moreover, if the weights are initially small but non-symmetric, mSTDP learning will eventually make them approximately symmetric [56]. This symmetry is assumed in many neural network models (from Hopfield [57] onward), but here we have shown how it can arise naturally from biologically realistic assumptions. We note that these scaled weights will lead to to a scaled feedback reconstruction, as described earlier, and identify $\alpha \approx \frac{\eta }{\zeta }$. In our simulations, for the first experiment we use the approximation that symmetric plasticity has already led to symmetric weights, and henceforth apply the substitution $Q\to \frac{\eta }{\zeta }W^{⊺}$, but in the second experiment we initialize **W** and **Q** separately and measure how quickly they become symmetric.

#### Spiking network implements scaled autoencoder learning rule

To begin our analysis of the effects of the mSTDP learning rule, we consider the timing of the three bouts of activity in our network (Fig 1b). We note that early visible layer spikes occur before hidden layer spikes, while late visible layer spikes due to feedback occur after the hidden layer spikes. Defining *S*_*i*,E_ and *S*_*i*,L_ as the sets of early and late visible layer spikes, respectively, and *S*_*j*_ as the set of hidden layer spikes, the mSTDP learning rule becomes:

$$\frac{1}{\eta }\Delta w_{ij}=\sum_{k\in S_{i,E}}\sum_{l\in S_{j}}e^{-(t_{l}-t_{k})/\tau_{+}}-\sum_{k^{\prime }\in S_{i,L}}\sum_{l^{\prime }\in S_{j}}e^{-(t_{k^{\prime }}-t_{l^{\prime }})/\tau_{-}}.$$ (13)

We next approximate the time differences (*t*_*l*_ − *t*_*k*_) by the average time between the early and intermediate activity bouts, which we call Δ*t*_1_ (Fig 1b). We similarly approximate (*t*′_*l*_ − *t*′_*k*_) by the average time between the intermediate and late activity bouts, Δ*t*_2_; we can now move the exponential terms outside the sums. We recall that the spike counts in the three bouts are given by $\vec{x}$, $\vec{y}$, and $\hat{x}$, so we finally have

$$\frac{1}{\eta }\Delta w_{ij}\approx e^{-\Delta t_{1}/\tau_{+}}x_{i}y_{j}-e^{-\Delta t_{2}/\tau_{-}}{\hat{x}}_{i}y_{j}=(\beta x_{i}-\gamma {\hat{x}}_{i})y_{j}$$ (14)

for *β* = *e* ^−Δ*t*_1_/*τ*_+_^ and *γ* = *e* ^−Δ*t*_2_/*τ*_−_^. A similar result holds if we do not approximate the times of the spikes but instead integrate over the shape of their distribution: If the density of spikes in the first bout is given by *x*_*i*_ *d*_*x*_(*t*)*dt*, where *d*_*x*_(*t*)*dt* integrates to 1 and is zero for times outside the bout, and if we define similar densities for the other bouts, then the learning rule is $\frac{1}{\eta }\Delta w_{ij}\approx \left(\int_{0}^{\infty }d_{x}(t)e^{-t/\tau_{+}}\int_{0}^{\infty }d_{y}(t)e^{t/\tau_{+}}\right)\;x_{i}y_{j}-\left(\int_{0}^{\infty }d_{\hat{x}}(t)e^{t/\tau_{-}}\int_{0}^{\infty }d_{y}(t)e^{-t/\tau_{-}}\right)\;{\hat{x}}_{i}y_{j}$ This has the same form as Eq 14, except with different values for *β* and *γ*. In either case, our learning rule becomes

$$\frac{1}{\eta \beta }\Delta w_{ij}=\frac{1}{\zeta \beta }\Delta q_{ji}^{⊺}\approx (x_{i}-\frac{\gamma }{\beta }{\hat{x}}_{i})y_{j}^{⊺}.$$ (15)

If parameters are such that $\frac{\gamma }{\beta }=\frac{1}{\alpha }$, this is exactly proportional to the desired autoencoder learning ruley $\Delta W\;\text{=}\;(\vec{x}-\frac{1}{\alpha }\hat{\vec{x}}){\vec{y}}^{⊺}$.

Concluding, we have shown that biologically plausible plasticity rules for feedforward and feedback connections in our spiking network cause it to approximately follow the scaled autoencoder learning rule. With our simulations, we will examine whether this approximation does in fact allow the network to minimize reconstruction error.

#### Synaptic scaling for sparsity

In order to learn sparse representations, autoencoder networks require additional regularizations or constraints. Here, we use a very simple mechanism with a clear biological interpretation: throughout the course of training, we adjusted each hidden neuron’s responsiveness to synaptic inputs so that it would maintain a low target average activity rate. This can be seen as an implementation of the experimentally observed phenomenon known as synaptic scaling [35–37]. Different values of the target activity rate correspond to different levels of sparsity in the learned representation; when the target activity rate is high, most hidden neurons respond to any given stimulus and the representation is very distributed. By contrast, for low target activity rates, most hidden neurons respond only to a small fraction of stimuli, leading to a sparse representation.

### Simulation results in spiking networks

We numerically simulated a network of LIF neurons. Because of spiking neurons’ nonlinear responses to input, the variable time courses of activity in the network, and the exponential STDP rules, a LIF network does not exactly follow the scaled autoencoder learning rule given in Eq (9). Moreover, the autoencoder learning rule itself performs only an approximate gradient descent on the reconstruction error. Our numerical simulations allowed us to investigate whether the LIF network could minimize the autoencoder loss function while still maintaining sparsity. (S1–S7 Tables).

**Fig 3:**
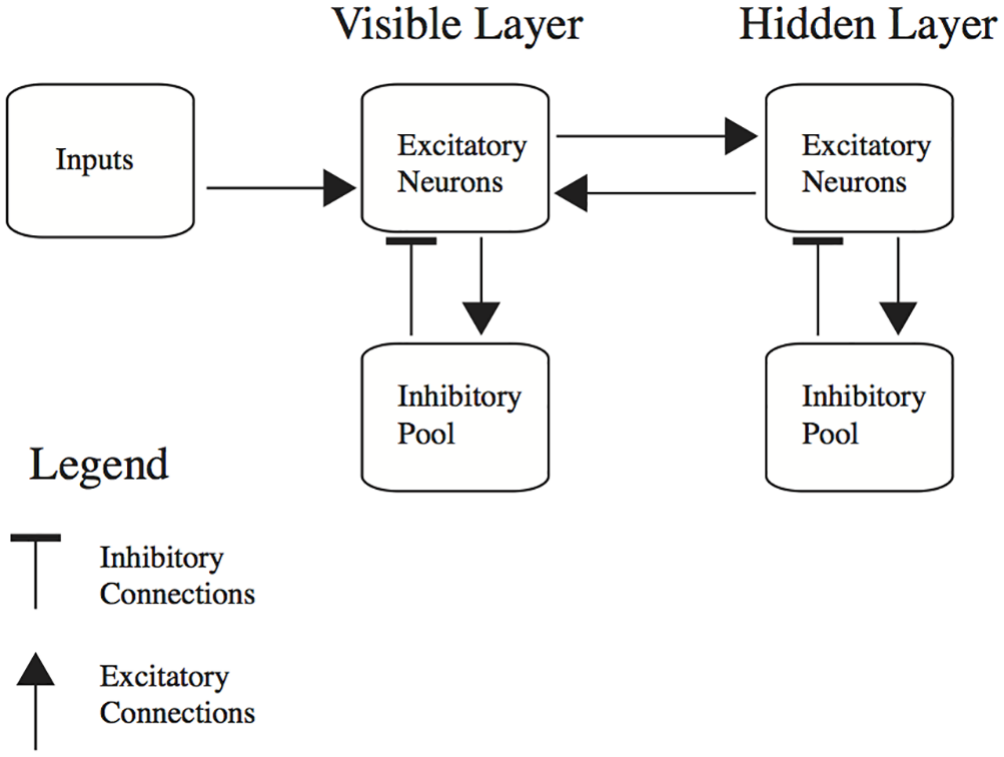
Model architecture for the integrate-and-fire simulations, including pools of inhibitory neurons in each layer.

The architecture of our simulated network was the same as that in Fig 1a, except that to control overall activity levels we included a pool of *N*_inh_ inhibitory neurons in each layer (Fig 3). The inhibitory neurons in each pool were connected reciprocally with every excitatory neuron in the layer. Connection weights to and from inhibitory neurons did not change during the simulations.

Our model neurons were conductance-based leaky integrate-and-fire neurons with a spike frequency adaptation term, similar to those in [58] (S6 Table). In our first experiment, where we trained the network with the MNIST dataset of handwritten numerals, synaptic weights from the visible and hidden units could take on positive or negative values. In our second experiment, where we trained the network with natural image patches, we imposed more biologically realistic constraints, and restricted weights from visible and hidden units to be positive only.

#### Synaptic scaling

To implement synaptic scaling in the MNIST experiment, we defined for each hidden neuron *j* a property *ϕ*_*j*_ that we called the “synaptic offset”. We used this to modify each of that neuron’s incoming synaptic connections, creating effective weights ${\tilde{w}}_{ij}=w_{ij}+\phi_{j}$. In practice, this approach is very similar to a threshold modification [7, 12] or to a standard neural network bias term, and it has consistent effects whether weights are positive or negative.

By contrast, in the natural image patches experiment, we implemented synaptic scaling with a multiplicative factor *Φ*_*j*_, for effective weights ${\tilde{w}}_{ij}=w_{ij}\times \Phi_{j}$. Because the weights were non-negative, neurons could consistently increase or decrease their net excitation, and thus their average activity, by increasing or decreasing this scaling factor.

Biological synaptic scaling typically has *multiplicative* effects on individual synaptic weights, but the scaling factors can differ between excitatory and inhibitory inputs [37], leading to additive as well as multiplicative effects on net synaptic inputs. Our synaptic offsets *ϕ*_*j*_ can be seen as modeling just the additive components of synaptic scaling, whereas the scaling factors *Φ*_*j*_ directly model multiplicative synaptic scaling on the excitatory weights.

During both experiments, we kept a running average *A*_*j*_ of the fraction of trials when each hidden unit fired at least one spike. We compared *A*_*j*_ to a target activation rate *ρ*, and after each trial changed *ϕ*_*j*_ or *Φ*_*j*_ according to Δ*ϕ*_*j*_ (or Δ*Φ*_*j*_) = *β*(*ρ* − *A*_*j*_) for learning rate *β* (S5 Table).

#### Training procedure

For the MNIST dataset, each of 50,000 training images was down-sampled to 14x14 pixels and the mean value across the training set was subtracted from each pixel before images are doubled to 392 ON/OFF input pixels. For each input, a Poisson train of input spikes was generated with mean rate equal to the pixel value of the input. Our network had 5,000 hidden units and was trained for two passes through the training set. We used a target activation rate *ρ* = 0.03, meaning that each hidden unit should fire at least one spike for approximately 3% of stimuli. For the second dataset, we used 16x16 pixel patches taken from the whitened natural images used in [16], which we mean-subtracted and doubled to 512 pixel inputs. We found that many of the patches had no high-contrast features; these patches activated the visible neurons only weakly and did not produce any activity in hidden units (and thus no learning.) For speeding up our simulations, then, we restricted training to high-contrast patches where the average value in the pre-processed patch was at least 0.06. We trained the network with 500 hidden units on 300,000 randomly selected high-contrast patches with a target activation rate of *ρ* = 0.02.

For each training stimulus, the network spiking response was calculated and feedforward and feedback weights were changed according to Eq 12. The learning rates were the same for feedforward and feedback weights. For the MNIST experiments, feedforward and feedback weights were initialized with symmetrical values, and Eq 12 exactly maintained this symmetry. For the more biologically realistic natural image patch experiments, the feedforward and feedback weights were independently initialized to random values.

#### Learned hidden unit receptive fields

**Fig 4:**
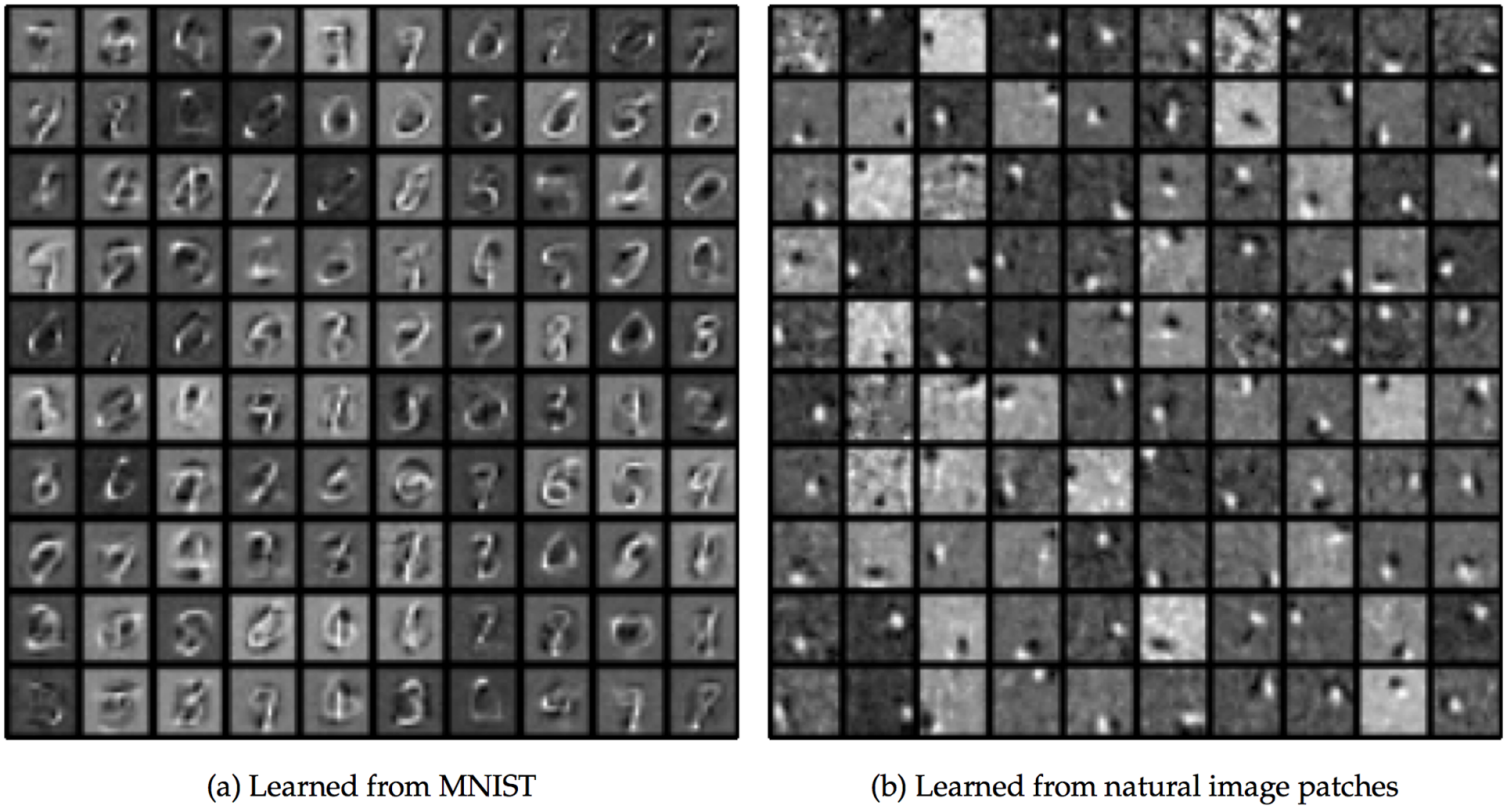
Feedforward weights after training for the MNIST and natural image patch datasets. **a:** Weights learned from the MNIST dataset. Each square in the grid represents the incoming weights to a single hidden unit; weights to the first 100 hidden units are shown. Weights from visible neurons which receive OFF inputs are subtracted from the weights from visible neurons which receive ON inputs. Then, weights to each neuron are normalized by dividing by the largest absolute value. **b:** Same as (a), but for the natural image patch dataset.

**Fig 5:**
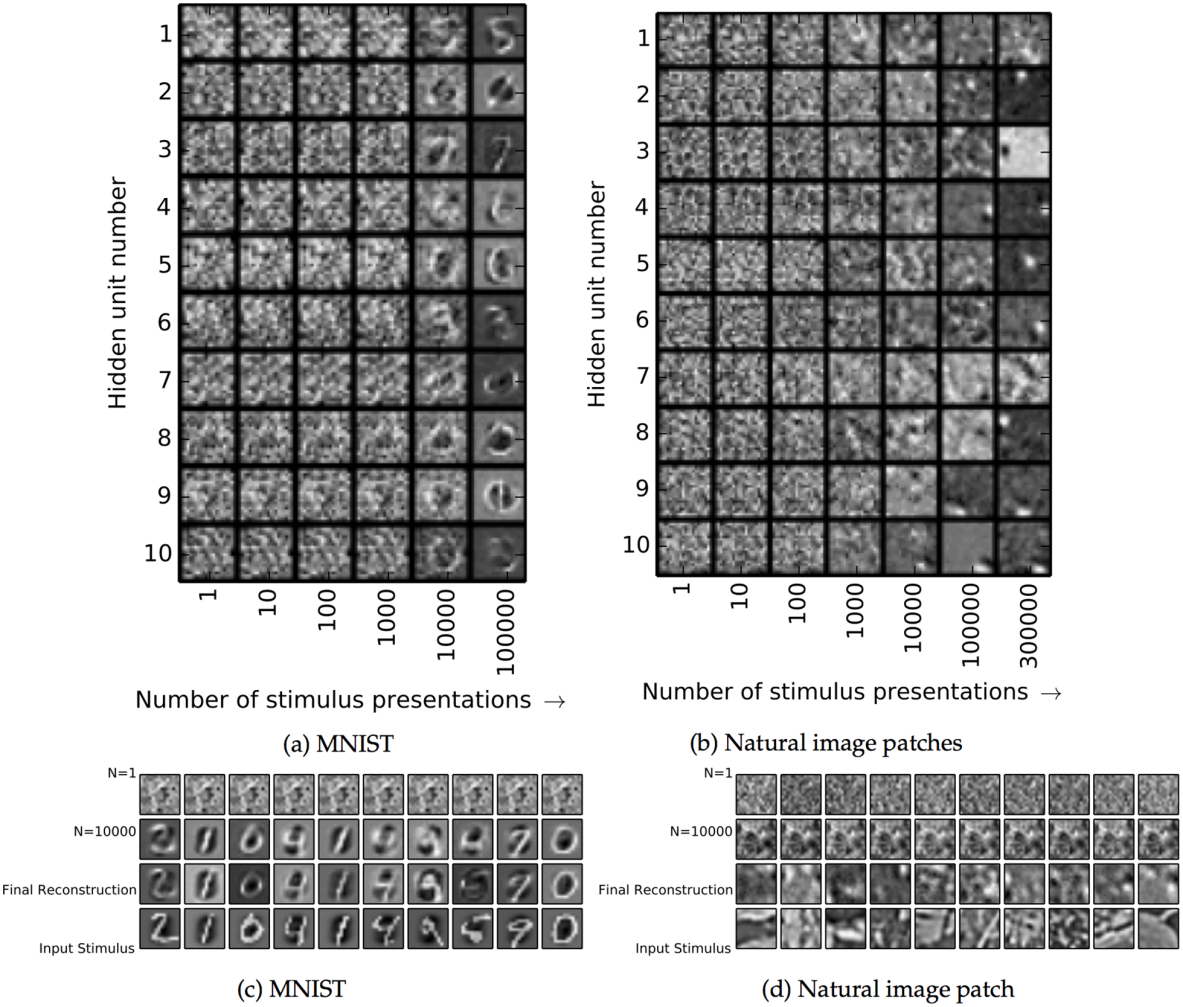
Evolution of weights and reconstructions in the spiking model. **a–b**: Evolution of weights in the spiking model. Weights as learned after different numbers of stimulus presentations are shown for 10 example hidden units. **c–d**: Attempted reconstructions at different points in training for the two spiking model experiments, for the stimuli shown in the bottom rows. Early in training, the same few hidden units whose incoming weights happened to be strongest were often activated regardless of the stimulus, leading to similar reconstruction attempts for different stimuli (first rows). Over time, the attempted reconstructions came to resemble the input stimuli.

In Fig 4a, we show the incoming weights *w*_*ij*_ or “receptive fields” for 100 out of the 5,000 hidden neurons in the MNIST network. For visualization, the weights from the visible cells with OFF inputs were subtracted from the weights from the visible cells with ON inputs. The network learned hidden-unit input weights with complex spatial structures (Fig 4a) somewhere between full digits and individual strokes. The development of the weights over time is shown in Fig 5, along with attempted reconstructions made with the weights at several different points in training. At the earliest time, the few hidden units that happened to have strong incoming weights would respond to any stimulus, making the different attempted reconstructions very similar.

In contrast to MNIST, the receptive fields learned by the natural image patch dataset (Fig 4b) were compact. They resembled the Gabor filters found in simple cells of primary visual cortex. Most receptive fields had two or more slightly elongated subregions receiving inputs from ON or OFF visible units.

#### Autoencoder performance

**Fig 6:**
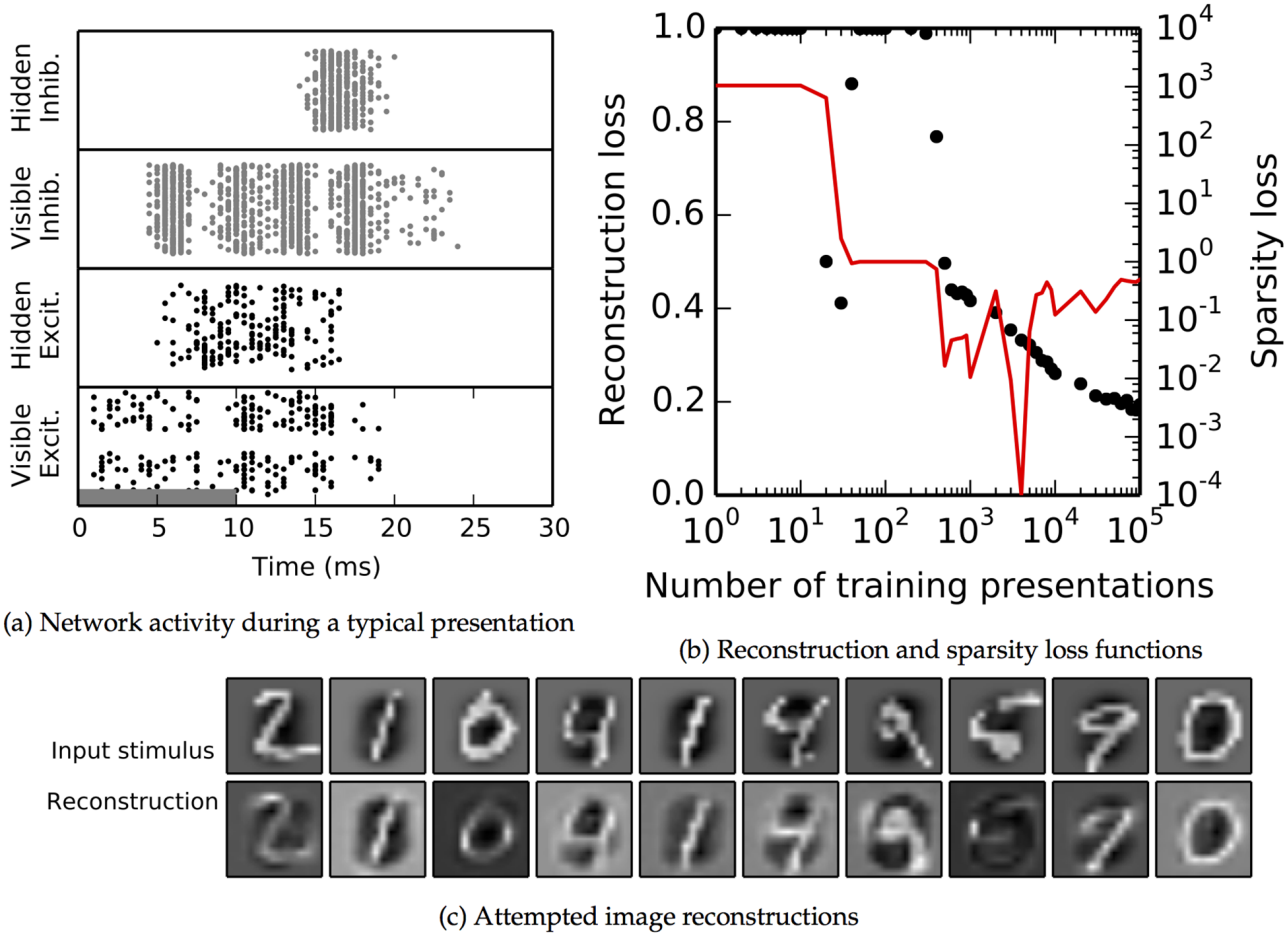
Behavior of the simulated spiking network for the MNIST dataset. **a:** Behavior of the network during a typical image presentation; compare with Fig 1b. Time period of external stimulation shown by grey bar. Raster plot includes all neurons which fired at least one spike during the presentation. The spikes of the visible neurons are in the bottom row and those of the hidden neurons are directly above. The top two rows, in grey, show the spikes for the inhibitory pools at each layer. Although the each training presentation ran for 65ms, all spikes occurred before 30ms so the raster plot was ended there. **b:** Reconstruction loss function, black dots, (defined in text) decreases over time, as does sparsity loss function (red, note log scale on y axis). **c**: The trained networks’ attempted reconstruction of representative training images. Each image shows the ON cell values minus the OFF cells. The first row shows the inputs to the network. The second row shows the attempted reconstruction $Q^{⊺}\vec{z}$.

Fig 6a shows an example raster plot of network activity for all the neurons that spiked during a single MNIST stimulus presentation after training was completed. (Compare with Fig 1b). There were 255 active visible neurons, 198 hidden ones, 1,175 active visible inhibitory neurons, and 962 hidden inhibitory neurons. The visible unit activity during the initial phase, from 0–10ms, was similar (but not identical) to that during the final reconstruction phase, from about 10–20ms. There were slightly fewer spikes in the later phase, corresponding to scaled feedback weights, but the general pattern is similar, indicating that the network has learned to reconstruct the inputs.

We note that the onset latency for hidden unit activity was between 5 and 10ms, and that most hidden units which produced spikes did so near the end of the initial bout of visible unit spikes. This onset delay was slightly shorter than the latency differences between visual areas in the primate visual system [59], and was not due to the synaptic transmission delay, which was 2ms; instead, the delay occurred because the network had learned feedforward weights which were weak enough that hidden units needed to integrate many incoming spikes before they could fire. These weak feedforward weights were maintained because any hidden units with strong incoming weights would fire earlier, while initial visible unit activity was ongoing; initial visible spikes occurring after the hidden unit spikes would cause depression instead of potentiation, and the hidden unit’s incoming weights would be weakened in the future.

To quantify how the network’s reconstruction ability changed over the course of training, we periodically disabled plasticity and calculated the network’s response to the same 100 test images. For each test presentation, we recorded the network inputs $\vec{\nu }$ and calculated $\vec{z}$ as the number of hidden neuron spikes. We measured the network’s feedback excitation as $Q^{⊺}\vec{z}$, and calculated a reconstruction loss as:

$$\text{Reconstruction}\;\text{loss}=1-\left\langle \mathrm{corr}\left(\vec{\nu },Q^{⊺}\vec{z}\right)\right\rangle$$ (16)

We defined average hidden unit activity levels *A*_*j*_ = ⟨*y*_*j*_ > 0⟩, where the average was taken across all test stimuli. We then defined a sparsity loss function as:

$$\text{Sparsity loss}={\left\|A_{j}-\rho \right\|}^{2}/\rho^{2}$$ (17)

In Fig 6b, we plotted both of these losses. The network quickly improved its reconstruction ability, arriving at reconstructions that were on average 80% correlated with their inputs. Meanwhile, lifetime sparsity was closely maintained. We conclude that the network successfully minimized its reconstruction error.

Fig 6c shows reconstructions for 10 representative input stimuli at the network’s final trained weights. The first row shows the input stimulus and the second row shows $Q\vec{z}$ (always with the OFF unit values subtracted from the ON unit values.) In all cases, the reconstructions closely resembled the inputs.

**Fig 7:**
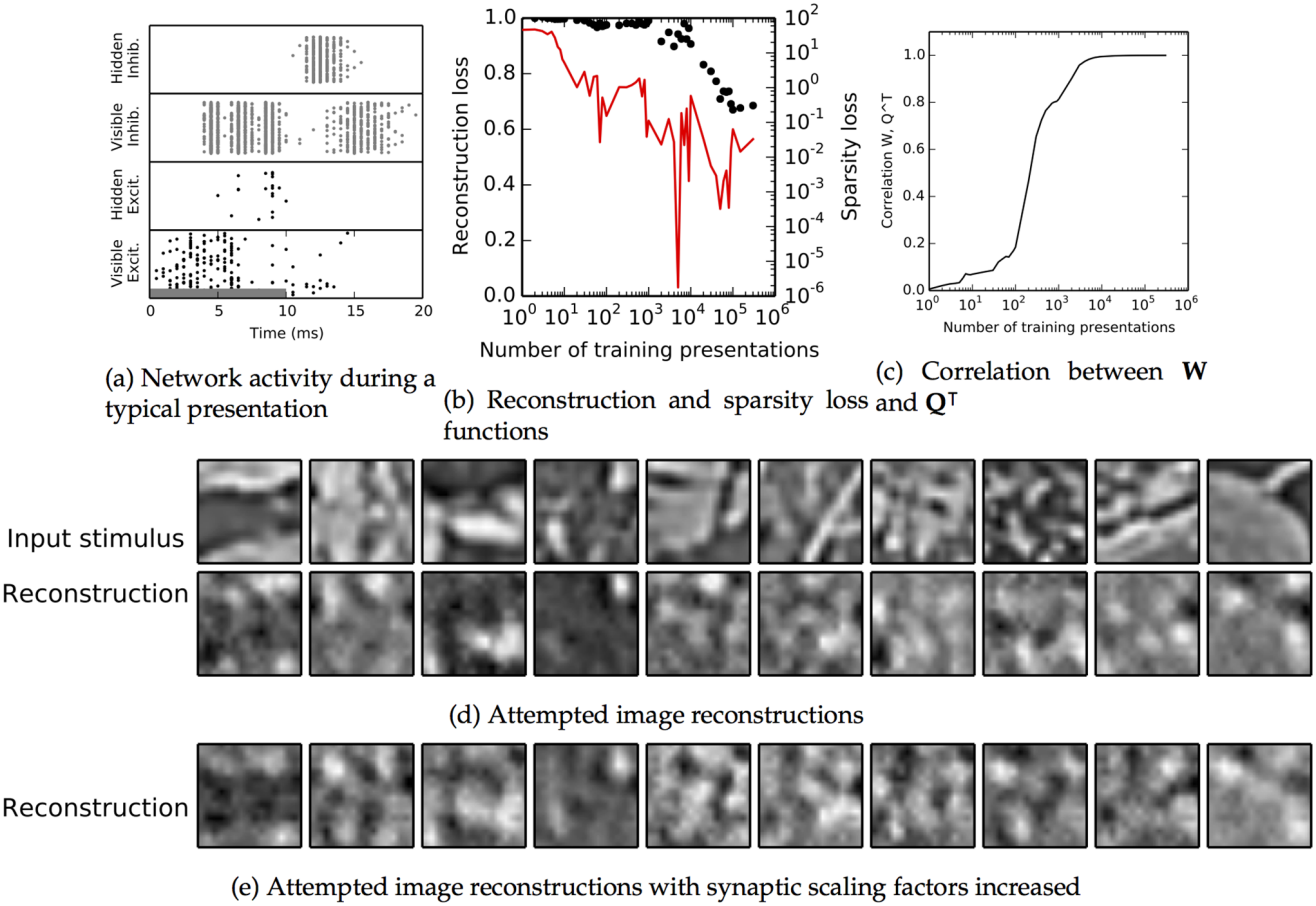
Behavior of the simulated spiking network for the natural image patch dataset. **a–d:** As Fig 6, with addition of **c**, which shows the Pearson correlation coefficient between the feedback weights **Q** and the feedforward weights **W** as they become symmetric over time. Because of the sparsity constraint on learning, the network cannot learn a perfect representation, so final autoencoder loss is still quite large (b), but the network nevertheless captures many salient features when attempting reconstruction (d). **e**: Reconstruction attempts for the same input stimuli as in (d), when the scaling factors Φ_*j*_ were uniformly multiplied by 1.5 after training.

We obtained similar results for the natural images dataset (Fig 7), albeit with substantially decreased final reconstruction performance. In the example presentation shown in the raster plot (Fig 7a), there were 311 active visible neurons, 25 hidden ones, 1,692 active visible inhibitory neurons, and 981 hidden inhibitory neurons. Initial visible unit spiking stopped while the input was still active, due to the influence of inhibition and spike frequency adaptation. The main difference, as compared to the MNIST dataset, is that the correlation between the input and the reconstruction remains at just below 35% (Fig 7b). The attempted reconstructions in Fig 7d captured many of the important features of the inputs but differed in the details. Light and dark areas in the reconstruction generally correspond to similar features in the stimulus, but many fine features, in particular thin and elongated features, are missed. Consequently, stimuli with wide features, such as the third stimulus in Fig 7d, are reconstructed with considerable success, while the network fails to adequately reconstruct the sixth stimulus in this Fig., which contains a prominent narrow line. Reconstruction performance could be improved slightly by increasing excitability in the network after training. We tried increasing each hidden unit’s scaling factor *Φ*_*j*_ by 50% during our reconstruction testing steps. This allowed more hidden neurons to become active during each presentation, including some which would otherwise have received only sub-threshold excitation. The activation of additional hidden units allowed reconstruction performance to improve visually in some cases (Fig 7); for instance, the curve in the upper-right-hand corner of the final stimulus is more fully traced out, and the dark band in the center of the first stimulus is more filled in. But the additional excitation did not fully resolve the difficulties in reconstruction; for example, in the second-to-last stimulus, the network is only able to reproduce about half of the black diagonal band across the center, and the reconstruction in the sixth stimulus is still poor. Quantitatively, correlations increased from between 1%-5% when tested at different points in training (S1 Fig).

#### Network learns distributed representations

**Fig 8:**
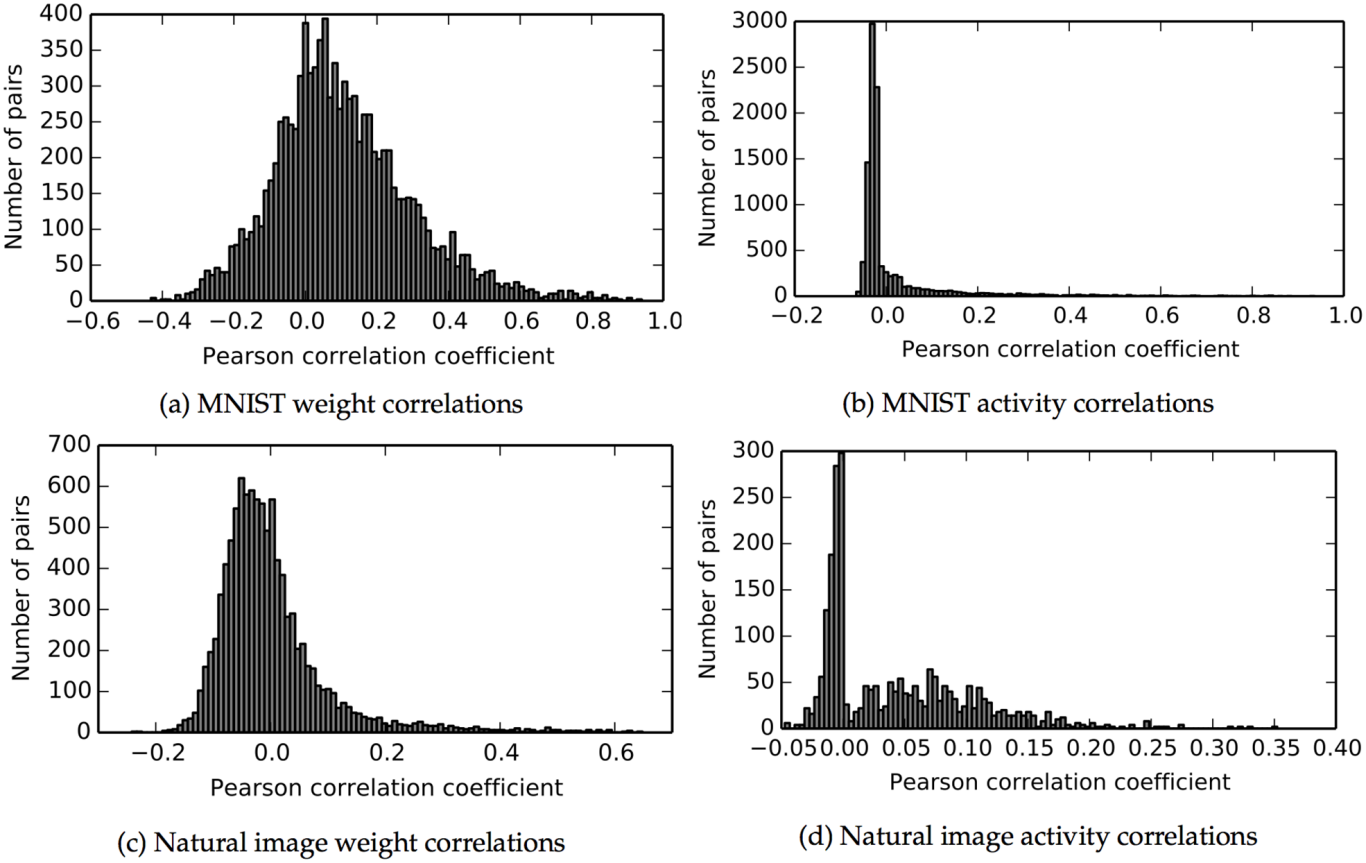
Hidden unit correlations after training. Neither the incoming weights nor the spiking activity is uncorrelated between hidden units. **a:** Correlations of the final trained synaptic weights between every pair of hidden units in the MNIST network. **b:** Correlations of the spike numbers from 1,000 stimulus presentations between every pair of hidden neurons for MNIST. **c–d:** Same, for the natural image network.

We have argued that an important strength of autoencoders is their ability to learn distributed representations without requiring the hidden units to be uncorrelated. To show that our network is not in the uncorrelated regime, we studied the stimulus-dependent correlations of the hidden unit receptive fields and activity. Fig 8a shows a histogram of the Pearson correlation coefficients between the vectors of incoming weights for each pair of hidden units in the trained MNIST network. Some pairs had positive correlations, indicating that the neurons could be excited by similar stimuli, while other pairs had negative correlations, meaning they would be unlikely to be activated at the same time. We confirmed that this implied correlated firing rates by measuring the responses of hidden units to 1,000 stimulus presentations, and calculating the Pearson correlation coefficients between the two vectors of spike counts for each neuron pair (Fig 8b). Because firing rates could not go below zero, this distribution was biased towards positive values. Fig 8c and 8d show similar results for natural image patches. We thus confirmed that our algorithm neither requires nor enforces hidden unit decorrelation.

**Fig 9:**
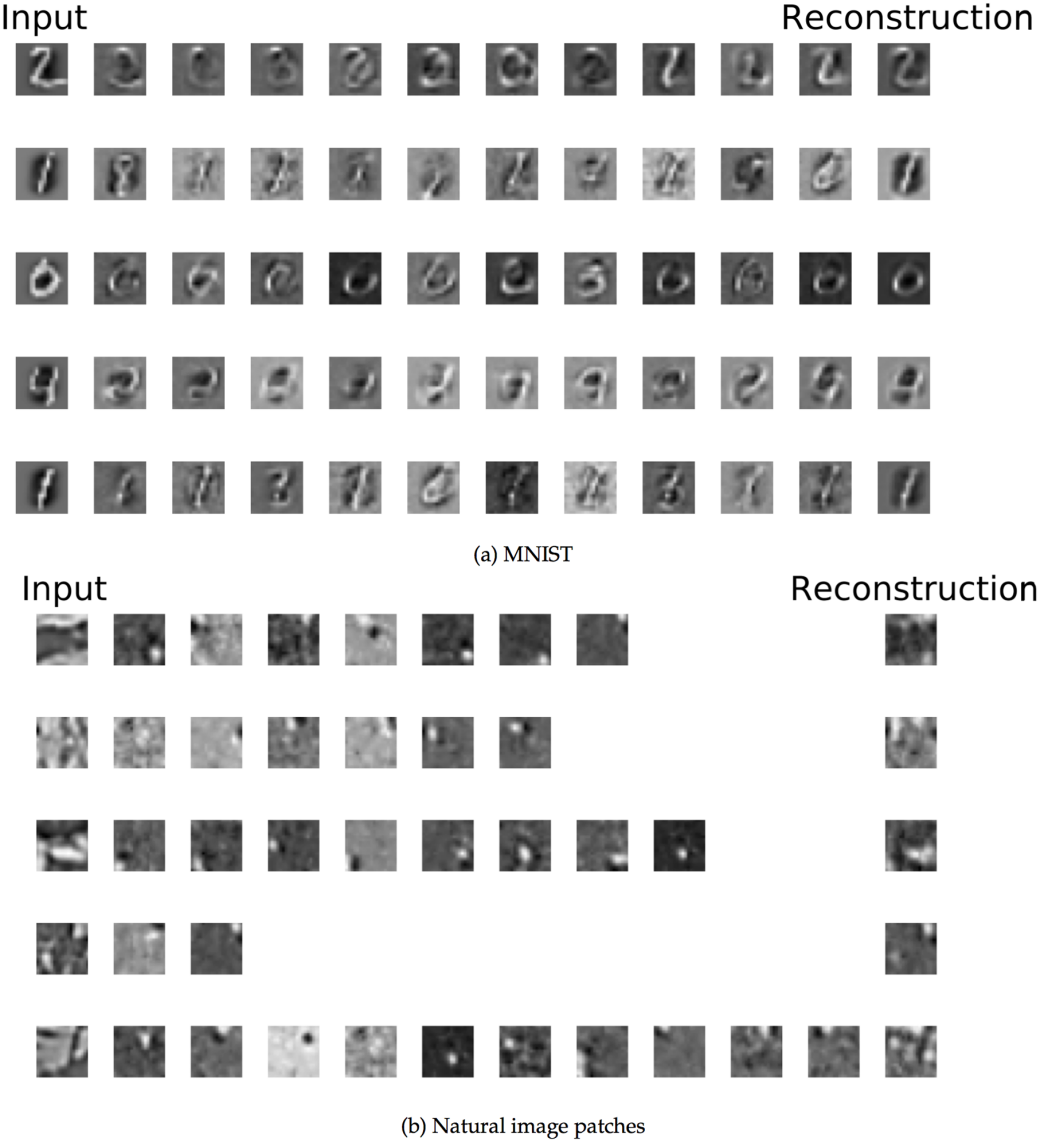
Highly activated weights for example image presentations in the two datasets. **a:** Weights for the MNIST dataset. First column shows 5 example inputs. Next 10 columns show the receptive fields of the 10 hidden units most activated for that input. Opacity codes the relative strength of the hidden unit’s responses with respect to the most active hidden unit; weakly activated hidden units are drawn nearly transparent. Final column shows the network’s attempted construction as measured by the late-time spike count. **b:** Same as (a), but for the the natural image patch dataset.

Fig 9 shows how multiple hidden units in our networks jointly represent each stimulus input. Using 5 example input stimuli for each of our two datasets, we selected the 10 hidden units which fired the most spikes in response to that input. We plotted the receptive fields for these hidden units in order, with the most strongly activated on the left. The reconstruction, calculated from all hidden units, is shown in the final column.

For MNIST (Fig 9a), some stimuli were well-matched by a single unit’s receptive field (for example, the bottom-most digit “1” stimulus.) However, others were not well-matched, and instead activated many hidden units. The “4” in the fourth row activated many hidden units, each of which differed from the input stimulus, but which nevertheless jointly created a very good reconstruction. This ability is dependent on the existence of a correlated representation; for instance, the first two hidden units in the third row have very similar receptive field structures and are likely to frequently be activated for the same stimuli.

For the natural image dataset (Fig 9b), the network strung together the Gabor-like receptive fields to represent stimuli. Here, because the receptive fields are more spatially localized, the hidden units activated for each stimulus did not typically have overlapping receptive fields. However, this does not mean that the different hidden units had uncorrelated firing responses across stimulus presentations. Indeed, particular groups of hidden units can be frequently co-active even though their receptive fields do not overlap. Consider the last four hidden units in the third row. Combined, these units’ receptive fields form a curve, a white “u” shape on a black background which resembles that seen near the bottom of the input stimulus. In natural images, elongated or curved structures like these are likely to occur relatively frequently, meaning that these four units might often be co-activated, leading to increased pairwise and higher-order correlations between these units. These correlations provide a signal that could be potentially learned by another layer of neurons; for instance, a neuron which learned to respond strongly to these four hidden units would be a curve detector.

#### The effect of changing the target activity level

**Fig 10:**
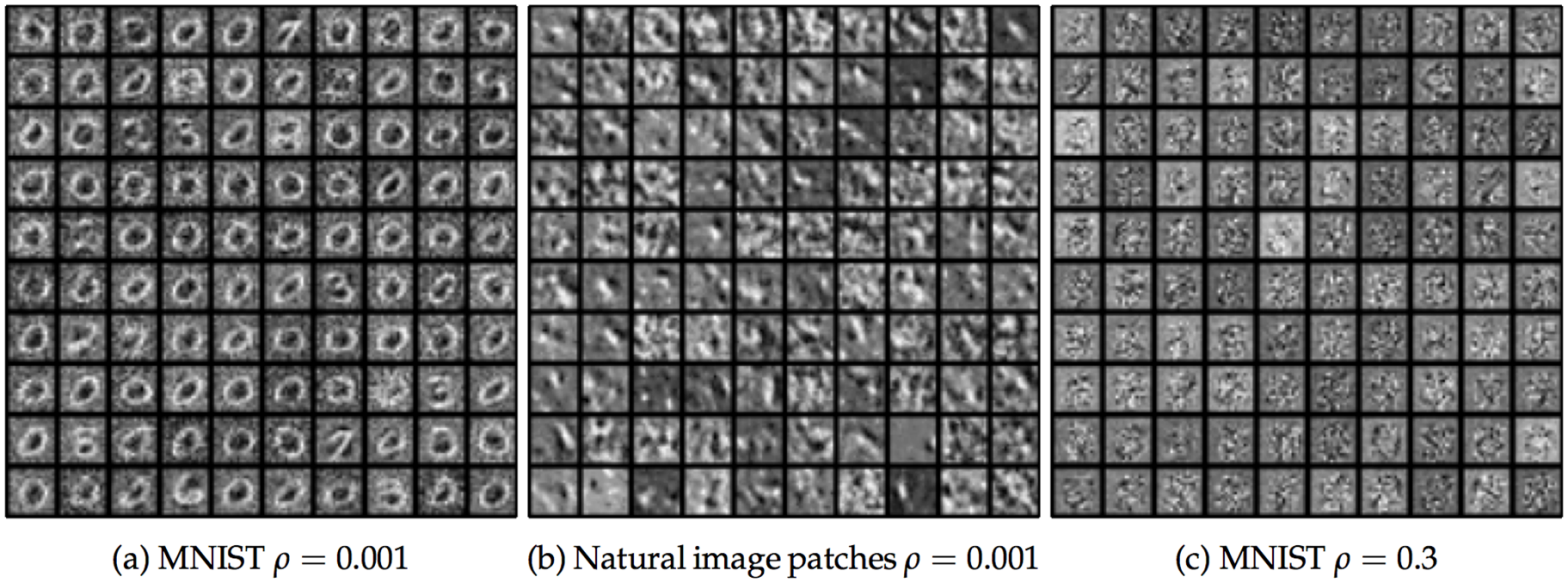
Learned hidden unit weights for different target activation rates *ρ*. **a:** Learned weights for the MNIST dataset with *ρ* = 0.001. **b:** Learned weights for the natural image patch dataset with *ρ* = 0.001. **c:** Learned weights for the MNIST dataset with *ρ* = 0.3.

The parameter *ρ* determined the level of sparsity in the learned representations, and thus was expected to greatly affect the forms of the hidden unit receptive fields. The results shown above were from simulations with *ρ* = 0.03 and *ρ* = 0.02, for the MNIST and natural image patch datasets, respectively. This corresponded to each hidden unit being active in about 3% or 2% of the stimulus presentations, respectively, which is significantly less than the fraction of neurons in early visual areas seen experimentally to respond to given stimuli [14]. Learned hidden unit receptive fields for different values of *ρ* are shown in Fig 10. For *ρ* = 0.001, corresponding to an even more sparse solution, hidden neurons tended to learn individual receptive fields that had larger support (Fig 10a and 10b). However, Fig 10a illustrates a potential shortcoming of the use of synaptic scaling to create sparsity: it can only control “lifetime sparseness” rather than “population sparseness” [60]. In Fig 10a, many hidden units have receptive fields that resemble the digit 0. Each individual unit fires very infrequently, but when a stimulus resembling a 0 appears, many units fire at the same time. To achieve true population sparsity, a model with some form of lateral interaction between hidden units would be needed. By contrast, high values of *ρ* meant that many hidden units would work together to represent each stimulus, so there was no requirement that individual receptive fields resemble any part of the stimulus. The MNIST network with *ρ* = 0.3 learned very distributed representations without clear structure to the receptive fields (Fig 10c). This network performed even better on the reconstruction task than the network with *ρ* = 0.03 in the main results, achieving a final reconstruction correlation of 91% compared with 80% for the main results. The natural image patch experiments, where weights were constrained to be non-negative, had difficulties with runaway excitation, and training results are therefore not available.

## Discussion

In this work, we propose a detailed and biologically realistic model for how spiking neurons could implement the commonly used unsupervised autoencoder learning algorithm. Our work provides a necessary first step in making biologically realistic models for any of the many unsupervised learning algorithms which include an autoencoder term, ranging from those inspired by machine learning to those inspired by biology, such as Sparse Coding [16]. We describe how strong feedforward and weak feedback excitation can drive a pattern of spiking activity that corresponds to the autoencoder’s visible unit input, hidden unit activity, and attempted reconstruction. Given this activity pattern, we show how STDP, a biological learning rule with strong experimental evidence, will cause changes in feedforward synaptic strength that approximate those dictated by the autoencoder learning rule. We argue that pure Hebbian STDP does *not*, however, cause the correct changes for the feedback synapses given this activity pattern. Instead, we draw upon recent experimental evidence to argue that those feedback synapses might learn according to a temporally reversed version of the learning rule, aSTDP, and show how STDP and aSTDP combine in the two-layer network context to form a symmetric learning rule we call mirrored STDP, or mSTDP. Finally, we show how mSTDP can allow both feedforward and feedback synapses to correctly implement the autoencoder learning rule.

We further describe how the network can find sparse representations by requiring its hidden units to fire infrequently. We argue that biological neurons could accomplish this through the experimentally observed process known as synaptic scaling.

This constraint was chosen here for its simplicity, but other forms of regularizers or sparsity constraint would also be compatible with our mirrored STDP model. For instance, in the Olshausen & Field Sparse Coding algorithm [16], hidden units in each trial find an optimal sparse steady-state through inhibitory lateral interactions and a term that could be modeled as spike rate adaptation. Once this steady-state is achieved, synaptic plasticity proceeds according to the autoencoder learning rule and could therefore be implemented with a model similar to ours.

Although we show here how networks could use mirrored STDP to implement autoencoder learning, we note that the basic principle can work independently of the specific plasticity mechanism. It only requires two factors. First, the network should have both a sensory-driven feedforward phase and feedback-driven attempted reconstruction phase. Second, during the feedforward phase, correlated firing should increase synaptic strength for both feedforward and feedback connections; in contrast, during the feedback phase, correlated firing should *decrease* synaptic strength. In our model, the decrease in synaptic strength during the feedback phase occurs because of the relative timing of activity in this phase. But similar results could be obtained, for instance, in a spike frequency model of plasticity in which weak firing due to feedback leaves neurons in a depressive regime (e.g. [61]).

Several previous spike-timing-based models of unsupervised feature learning have been successful at learning receptive fields that resemble those seen in V1; these include Rank Order Coding using SpikeNET, by Delorme, Perrinet and Thorpe [4], and the SAILNet model of Zylberberg and colleagues [12]. Neither network can learn a dense distributed code: in Rank Order Coding, only a single hidden unit responds to each local stimulus, while in SAILNet, hidden units are encouraged to be uncorrelated and fire very infrequently. Non-distributed, biologically realistic models have even successfully been extended into mid-level visual areas; for instance, Masquelier and Thorpe have shown that a winner-take-all STDP model was capable of learning good features in the second level of a max-pooling hierarchy [5]. We argue that distributed representations are likely to be better models for yet higher visual areas because of their increased representational capacity. However, additional work will be required to elucidate the conditions under which distributed representations—such as those which can be learned by the autoencoder—are warranted, and when the simpler learning mechanisms used in winner-take-all networks will suffice.

We test our model using two-layer networks of simulated integrate-and-fire neurons using two datasets: handwritten digits in the MNIST dataset and whitened natural image patches. In both cases, the network learns distributed hidden unit representations which are capable of reconstructions. However, the reconstruction performance is not as good for the natural image patches as for the MNIST dataset. This may in part be due to the fact that our current implementation only allowed us to explore the extremely sparse regime with low hidden unit activity, since parameters that led to less sparse solutions caused difficulties with runaway excitation during training. Indeed, when we increased the network activity after training by manually increasing synaptic scaling factors by 1.5, reconstruction performance improved (Fig 7). Future work will be required to elucidate whether the training principles described here would continue to function in a more complicated network that is more robust to runaway excitation.

In the case of the natural image patches, the learned feedforward weights resemble those observed in the early mammalian visual system. As such, the autoencoder may be a useful model to consider when studying the development of connections between pyramidal neurons in the lateral geniculate nucleus and primary visual cortex, or between primary and secondary visual cortex. Of course, early visual areas of the brain cannot learn different sets of receptive fields for different stimuli, as in the two datasets we used here. They must learn very general representations that can be used to build more specific representations further up in the cortical hierarchy. However, in those higher brain areas, if specific sets of neurons are activated in response to different types of stimuli (such as faces), it is conceivable that an autoencoder-like algorithm could allow the development of more specialized receptive fields.

By restricting our model neurons in the natural image patch experiments to have non-negative weights, we show that autoencoder learning can work when the neurons follow Dale’s law. However, a true understanding of how sparse response patterns can arise will require a model for the development of selective inhibition. Neurons with purely excitatory receptive fields can exhibit sparse firing when those receptive fields are very small or the excitation very weak. Indeed, the receptive fields learned by our model neurons with the natural image patches were localized to small regions. By contrast, when receptive fields can have inhibitive components, as in our MNIST experiment, neurons can fire in a sparse manner even when the receptive fields are large and complex. Future work is needed to explore how plasticity in inhibitory neurons might help develop these complex receptive fields.

## Supporting Information

S1 Fig Reconstruction losses for natural images, calculated using unscaled (dashed line) feedforward weights versus those scaled by a factor of 1.5 (solid line). Reconstruction losses were calculated for the weights as learned at different points in the training. Weights were always unscaled during the training process, and scaling was applied only during the measurements of reconstruction loss.
(TIFF)

S1 Table Model summary table. (PDF)

S2 Table The neuronal populations present in the model. (PDF)

S3 Table The connectivity between different neuronal populations. (PDF)

S4 Table The inputs used to train the neural networks. (PDF)

S5 Table The equations describing plasticity in the network. (PDF)

S6 Table The equations describing the internal dynamics of model neurons. (PDF)

S7 Table The specific parameters used in the simulations described in the text. (PDF)
