# Supplementary figures and images for: Mirrored STDP Implements Autoencoder Learning in a Network of Spiking Neurons
Source: PLoS Comput Biol. 2015 Dec 3;11(12):e1004566. doi: 10.1371/journal.pcbi.1004566 (PMC4669146; doi:10.1371/journal.pcbi.1004566)

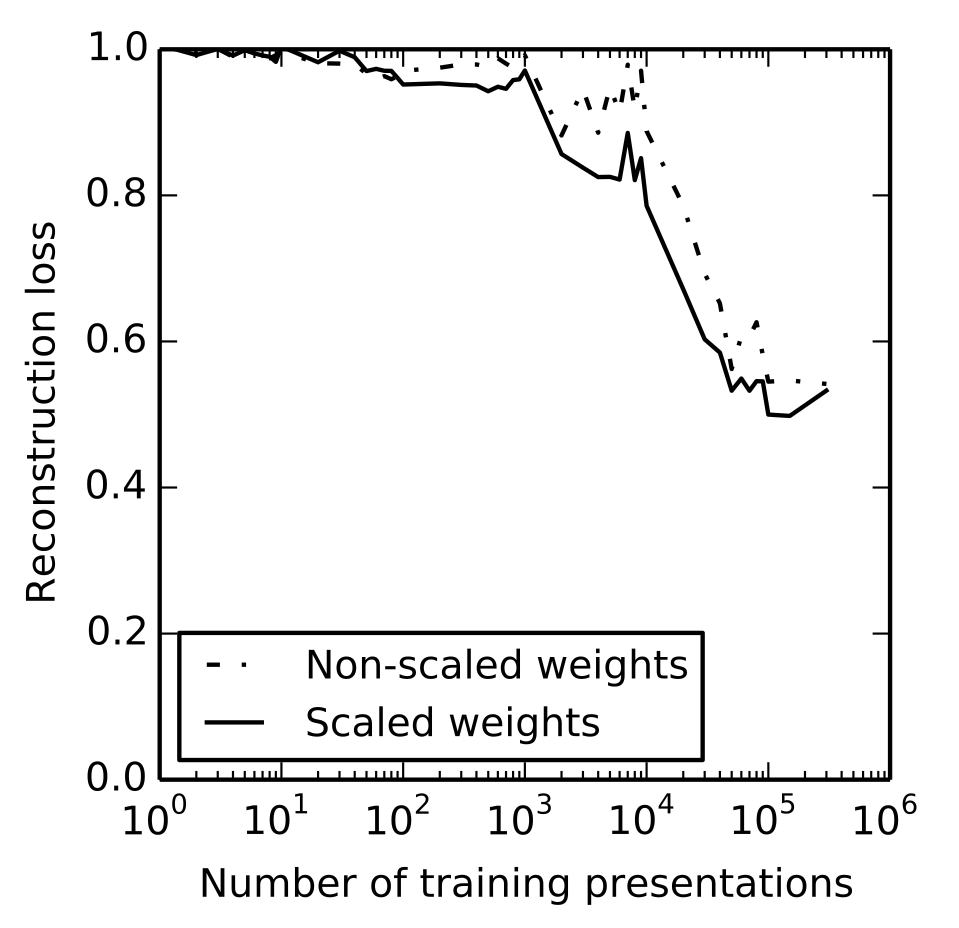

Supplement: S1 Fig — Reconstruction losses were calculated for the weights as learned at different points in the training. Weights were always unscaled during the training process, and scaling was applied only during the measurements of reconstruction loss. (TIFF) [file pcbi.1004566.s001.tiff]
