# Supplementary material for: Mirrored STDP Implements Autoencoder Learning in a Network of Spiking Neurons
Source: PLoS Comput Biol. 2015 Dec 3;11(12):e1004566. doi: 10.1371/journal.pcbi.1004566 (PMC4669146; doi:10.1371/journal.pcbi.1004566)
Supplement: S1 Table — (PDF) [file pcbi.1004566.s002.pdf]

S1 Table. Model Summary

**Populations**	Four: Visible and Hidden neurons, and one Inhibitory Pool for each layer.
**Connectivity**	Each visible unit connects reciprocally to each hidden unit. No lateral connections. Each visible and hidden unit connects reciprocally to every inhibitory unit in that layer's pool.
**Neuron model**	Leaky integrate-and-fire with synaptic scaling and spike-frequency adaptation.
**Synapse model**	Conductance inputs with instantaneous rise after transmission delay and exponential decay.
**Input pre-processing**	Mean-value subtraction, whitening (natural image patches only), rectification & concatenation to ON/OFF units
**Training**	For N_{max} repetitions:<br>1. Choose input stimulus. 2. Set $t = 0$ and reinitialize all neurons to random membrane potentials. 3. Apply input stimulus until $t = t_{\mathrm{stim}}$. 4. Turn off stimulus, continue running network until $t = t_{\mathrm{max}}$. 5. Calculate and apply weight changes.
**Plasticity**	Weights via mSTDP, synaptic scaling via homeostatic adaptation
**Measurements**	Learned weights, reconstruction error, hidden unit correlations.
