# Supplementary material for: Mirrored STDP Implements Autoencoder Learning in a Network of Spiking Neurons
Source: PLoS Comput Biol. 2015 Dec 3;11(12):e1004566. doi: 10.1371/journal.pcbi.1004566 (PMC4669146; doi:10.1371/journal.pcbi.1004566)
Supplement: S2 Table — (PDF) [file pcbi.1004566.s003.pdf]

S2 Table. Populations.

**Name**	Kind	Size
Visible	Leaky integrate and fire	$N_{\text{Vis}} = 2N_{\text{pixels}}$
Hidden	Leaky integrate and fire	N_{Hid}
Inhibitory	Leaky integrate and fire	$N_{\text{Vis,Inh}}, N_{\text{Hid,Inh}}$
