# Supplementary material for: Mirrored STDP Implements Autoencoder Learning in a Network of Spiking Neurons
Source: PLoS Comput Biol. 2015 Dec 3;11(12):e1004566. doi: 10.1371/journal.pcbi.1004566 (PMC4669146; doi:10.1371/journal.pcbi.1004566)
Supplement: S3 Table — (PDF) [file pcbi.1004566.s004.pdf]

S3 Table. Connectivity

**Name**	**Source**	**Target**	**Pattern**
Feedforward	Visible	Hidden	All-all, weights $\mathbf{W}$, synaptic scaling factors $\vec{\phi}$ or Φ, delay D
Feedback	Hidden	Visible	All-all, weights $\mathbf{Q}$, delay D
To Visible Inhibitory	Visible	Visible inhibitory pool	All-all, weights $\mathbf{W}_{\mathrm{Vis,Inh}}$, delay D
From Visible Inhibitory	Visible inhibitory pool	Visible	All-all, weights $\mathbf{W}_{\mathrm{Inh,Vis}}$, delay D
To Hidden Inhibitory	Hidden	Hidden inhibitory pool	All-all, weights $\mathbf{W}_{\mathrm{Hid,Inh}}$, delay D
From Hidden Inhibitory	Hidden inhibitory pool	Hidden	All-all, weights $\mathbf{W}_{\mathrm{Inh,Hid}}{}^{\intercal}$, delay D
