# Supplementary material for: Mirrored STDP Implements Autoencoder Learning in a Network of Spiking Neurons
Source: PLoS Comput Biol. 2015 Dec 3;11(12):e1004566. doi: 10.1371/journal.pcbi.1004566 (PMC4669146; doi:10.1371/journal.pcbi.1004566)
Supplement: S4 Table — (PDF) [file pcbi.1004566.s005.pdf]

S4 Table. Inputs

Type	Description
ON/OFF inputs	Begin with N_{pixels} pixel values $\nu_{\text{ext},k}$ for images selected from training sets of mean-subtracted MNIST images or whitened natural image patches. Generate $2N_{\text{pixels}}$ homogeneous Poisson input spike trains $\mathcal{S}_{\text{ON/OFF},i}$. The first N_{pixels} spike trains represent ON cells and have mean firing rates $\nu_i = \xi \times max(0, \nu_{\text{ext},i})$, while the second N_{pixels} spike trains represent OFF cells and have mean firing rates $\nu_i = \xi \times max(0, -\nu_{\text{ext},i-N_{\text{pixels}}})$. ξ is an input scaling factor. Spikes are generated only during the time interval $0 < t < t_{\text{max}}$. Each visible unit receives excitatory input from a single input spike train.
