# Supplementary material for: Mirrored STDP Implements Autoencoder Learning in a Network of Spiking Neurons
Source: PLoS Comput Biol. 2015 Dec 3;11(12):e1004566. doi: 10.1371/journal.pcbi.1004566 (PMC4669146; doi:10.1371/journal.pcbi.1004566)
Supplement: S5 Table — (PDF) [file pcbi.1004566.s006.pdf]

S5 Table. Plasticity

**Type**	**Description**
mSTDP	$\mathbf{W}$ and $\mathbf{Q}$ initialized separately, uniformly distributed in range $(0, W_{\text{init}})$. For visible unit i, hidden unit j, feedforward learning rate η: $\Delta w_{ij} = \Delta q_{ji} = \eta \sum_{k\in\mathcal{S}_i} \sum_{l\in\mathcal{S}_j} \begin{cases} +e^{-\lvert t_l - t_k\rvert/\tau_+} & \text{if } t_l > t_k \\ -e^{-\lvert t_l - t_k\rvert/\tau_-} & \text{if } t_l \leq t_k \end{cases}.$ Weights to-from inhibitory pools $\mathbf{W}_{\text{Vis,Inh}}$ initialized separately from exponential distributions with means $W_{\text{Vis,Inh}}$ etc, and do not undergo plasticity.
Homeostatic adaptation	Synaptic scaling factors initialized to ϕ_{init} or Φ_{init}. $\Delta\phi_j = \Delta\Phi_j = \beta(\rho - A_j)$ ρ = target activation rate, A_j = average activation, initialized equal to 0 and updated after each presentation via $A_j \leftarrow \begin{cases} e^{1/\tau_{\text{fr}}} A_j + \left(1 - e^{1/\tau_{\text{fr}}}\right) & \text{if neuron j active during the presentation} \\ e^{1/\tau_{\text{fr}}} A_j & \text{otherwise} \end{cases}.$
