# Supplementary material for: Mirrored STDP Implements Autoencoder Learning in a Network of Spiking Neurons
Source: PLoS Comput Biol. 2015 Dec 3;11(12):e1004566. doi: 10.1371/journal.pcbi.1004566 (PMC4669146; doi:10.1371/journal.pcbi.1004566)
Supplement: S6 Table — (PDF) [file pcbi.1004566.s007.pdf]

S6 Table. Neuron and Synapse Models

Name	Equations
Subthreshold dynamics	$$\begin{aligned}\tau_{\text{memb}}\dot{V}_i(t) &= (V_{\text{rest}} - V_i(t)) \\ &+ (V_{\text{ex}} - V_i(t)) \left(E_i(t) + E_{\text{ON/OFF},i}(t)\right) \\ &+ (V_{\text{inh}} - V_i(t))\, I_i(t) + (V_{\text{adap}} - V_i(t))\, I_{\text{adap},i}(t)\end{aligned}$$
Conductances	$\tilde{w}_{ij} = w_{ij} + \phi_j$ or $\tilde{w}_{ij} = w_{ij} \times \Phi_j$<br>Synaptic inputs: Excitatory inputs $E_i(t) = \tau \sum_j \sum_{l \in \mathcal{S}_j} \tilde{w}_{ij} s(t - (t_l + D))$; analogous for $E_j(t)$ and inhibitory inputs I_i<br>External inputs: $E_{\text{ON/OFF},i}(t) = \tau \sum_{l \in \mathcal{S}_{\text{ON/OFF},i}} s(t - t_l)$; for visible units only<br>Adaptation: $I_{\text{adap},i}(t) = \sum_{l \in \mathcal{S}_i} exp(-\frac{t - t_l}{\tau_{\text{adap}}}) \times (t > t_l)$<br>Synaptic profile: $s(t) = exp(-\frac{t}{\tau_\alpha}) \times (t > 0)$
Spiking	If $V_i(t) \geq V_{\text{thr}}$:<br>1. Add spike with time-stamp $t_k = t$ to set $\mathcal{S}_i$<br>2. Set $V_i(t) = V_{\text{rest}}$
