# Supplementary material for: Mirrored STDP Implements Autoencoder Learning in a Network of Spiking Neurons
Source: PLoS Comput Biol. 2015 Dec 3;11(12):e1004566. doi: 10.1371/journal.pcbi.1004566 (PMC4669146; doi:10.1371/journal.pcbi.1004566)
Supplement: S7 Table — (PDF) [file pcbi.1004566.s008.pdf]

S7 Table. Parameters

Symbol	Description	MNIST value	Natural Images value
N_{pixels}	Number of pixels in input images before pre-processing	196	256
N_{vis}	Number of visible units	396	512
N_{hid}	Number of hidden units	5000	500
$N_{\text{Vis,Inh}}$	Number of inhibitory units in visible layer	300	300
$N_{\text{Hid,Inh}}$	Number of inhibitory units in hidden layer	1000	1000
D	Synaptic transmission delay	2ms	2ms
W_{init}	Maximum initial feedforward and feedback weight	0.25	3
$W_{\text{Vis,Inh}}$	Average visible to inhibitive weight	0.5	1.0
$W_{\text{Hid,Inh}}$	Average hidden to inhibitive weight	0.1	1.0
$W_{\text{Inh,Vis}}$	Average inhibitive to visible weight	0.15	0.1
$W_{\text{Inh,Hid}}$	Average inhibitive to hidden weight	0.3	0.1
$\phi_{\text{init}}, \Phi_{\text{init}},$	Initial value of hidden unit synaptic scaling factor	0.2	0.05
η	Learning rate for feedforward connections	0.003	0.3
β	Learning rate for synaptic scaling factor	0.01	0.03
ρ	Target activation rate	0.03	0.02
τ_{FR}	Time constant for calculating average hidden activations	30 presentations	300 presentations
τ_{memb}	Membrane potential time constant	30ms	30ms
τ_{α}	Synapse time constant	2ms	2ms
τ_{+}	Timescale for mSTDP potentiation	30ms	30ms
τ_{-}	Timescale for mSTDP depression	60ms	60ms
t_{max}	Training presentation running time	65ms	65ms
ξ	Scale for inputs	10	15
V_{thr}	Spiking threshold potential	-54mV	-54mV
V_{rest}	Rest potential	-70mV	-70mV
V_{ex}	Excitatory reversal potential	0mV	0mV
V_{inh}	Inhibitory reversal potential	-80mV	-80mV
V_{adap}	Adaptation reversal potential	-80mV	-80mV
